# A biomimetic nanoplatform for customized photothermal therapy of HNSCC evaluated on patient-derived xenograft models

**DOI:** 10.1038/s41368-022-00211-2

**Published:** 2023-02-10

**Authors:** Qi Wu, Lan Chen, Xiaojuan Huang, Jiayi Lin, Jiamin Gao, Guizhu Yang, Yaping Wu, Chong Wang, Xindan Kang, Yanli Yao, Yujue Wang, Mengzhu Xue, Xin Luan, Xin Chen, Zhiyuan Zhang, Shuyang Sun

**Affiliations:** 1 Department of Oral and Maxillofacial-Head & Neck Oncology, Shanghai Ninth People’s Hospital, Shanghai Jiao Tong University School of Medicine; College of Stomatology, Shanghai Jiao Tong University; National Center for Stomatology; National Clinical Research Center for Oral Diseases; Shanghai Key Laboratory of Stomatology; Shanghai Research Institute of Stomatology, Shanghai, China; 2 Shanghai Frontiers Science Center for Chinese Medicine Chemical Biology, Institute of Interdisciplinary Integrative Medicine Research, Shanghai University of Traditional Chinese Medicine, Shanghai, China; 3 School of Chemical Engineering and Technology, Shaanxi Key Laboratory of Energy Chemical Process Intensification, Institute of Polymer Science in Chemical Engineering, Xi’an Jiao Tong University, Xi’an, Shaanxi China

**Keywords:** Oral cancer, Experimental models of disease

## Abstract

Cancer cell membrane (CCM) derived nanotechnology functionalizes nanoparticles (NPs) to recognize homologous cells, exhibiting translational potential in accurate tumor therapy. However, these nanoplatforms are majorly generated from fixed cell lines and are typically evaluated in cell line-derived subcutaneous-xenografts (CDX), ignoring the tumor heterogeneity and differentiation from inter- and intra- individuals and microenvironments between heterotopic- and orthotopic-tumors, limiting the therapeutic efficiency of such nanoplatforms. Herein, various biomimetic nanoplatforms (CCM-modified gold@Carbon, i.e., Au@C-CCM) were fabricated by coating CCMs of head and neck squamous cell carcinoma (HNSCC) cell lines and patient-derived cells on the surface of Au@C NP. The generated Au@C-CCMs were evaluated on corresponding CDX, tongue orthotopic xenograft (TOX), immune-competent primary and distant tumor models, and patient-derived xenograft (PDX) models. The Au@C-CCM generates a photothermal conversion efficiency up to 44.2% for primary HNSCC therapy and induced immunotherapy to inhibit metastasis via photothermal therapy-induced immunogenic cell death. The homologous CCM endowed the nanoplatforms with optimal targeting properties for the highest therapeutic efficiency, far above those with mismatched CCMs, resulting in distinct tumor ablation and tumor growth inhibition in all four models. This work reinforces the feasibility of biomimetic NPs combining modular designed CMs and functional cores for customized treatment of HNSCC, can be further extended to other malignant tumors therapy.

## Introduction

Photothermal therapy (PTT), a noninvasive therapy that does not suffer from restriction of chemo-resistance or radio-resistance, has demonstrated promising prospects for cancer treatment.^1,2^ In the case of superficial tumors such as head and neck squamous cell carcinoma (HNSCC), near-infrared (NIR) light responsive PTT is preferable due to effective photo penetration.^3–5^ Besides direct damage to cancer cells, PTT has also been shown to promote immunogenic cell death (ICD), a process that causes cancer cells to release tumor-associated antigens, which further trigger the anti-tumor immune response,^6,7^ generating the cytotoxic T lymphocytes (CTLs) to eliminate residual tumor cells and inhibit its metastasis.^8^

Until now, many PTT studies of oncotherapy based on various photothermal agents (PTAs) have been reported.^9–11^ Among these PTAs, gold (Au)-based nanomaterials play a critical role because of their localized surface plasmon resonance (LSPR) property.^12–17^ A clinical study also reported that Au nanomaterial-mediated focal laser ablation was successful for 94% (15/16) of prostate cancer patients without serious complications or deleterious changes in genitourinary function.^18^

Although the Au-based nanomaterials demonstrate an effective PTT approach, these nanomaterials still suffer from insufficient tumor accumulation because of their wide distribution in bio-systems via metabolic processes, which lead to the induction of incomplete tumor elimination and unpredictable side effects in normal tissues and organs.^19^ Additionally, owing to the complex physiological environment, the tumor targeting efficiency of these nanomaterials is still limited, even in the case of modified molecular targets.^20,21^ A survey verified that only ~0.7% of the administered nanoparticles (NPs) were found to be delivered to a solid tumor during therapy.^22^ Therefore, it is necessary to develop novel Au nanomaterials for effective photothermal tumor therapy and to overcome off-target side effects.

Recent advances in cancer biology have reported that intercellular interactions regulate intracellular signaling via contacts of cell membranes (CMs) in tumor growth and development.^23^ As a result of the functionalization of the homologous binding molecules on cancer CMs (CCMs), CCM-cloaked NPs have been shown to significantly promote cell endocytosis and homologous-targeting tumor accumulation in vivo.^24,25^ Therefore, Au nanomaterials coated with CCMs have gained increasing attention for tumor targeted PTT.^26–28^ However, most CCM-coated Au nanomaterials were developed based on tumor CMs of fixed cell lines or subcutaneous tumor models; this approach ignored heterogeneity and differentiation among tumor cells from inter- and intra-individuals as well as different tumor microenvironments between heterotopic- and orthotopic-tumor models, resulting in limited and inconsistent therapeutic efficiency in various in vivo models. Thus, the development of photothermal Au nanomaterials with modularly designed surfaces, which consists of specific CCM exactly matching the type and status of the tumor to be treated, is of significance for the applications of PTT to meet the requirements of different patients.

Herein, we developed a series of CCM-coated Au@Carbon core-shell NPs (Au@C-CCM) as customized therapeutic nanoplatforms, which were homologously tailored for anti-tumor therapy of specific HNSCC patients. The Au@C core served as PTA for tumor therapy. The CCM corona is responsible for the tumor-targeting property of the nanoplatforms and allowed the nanoplatform to selectively perform the photothermal conversion in tumor tissues for accurate treatment. During therapy, the Au@C-CCM was first targeted and accumulated in tumor tissues after tail vein injection due to the specific interaction between the tumor cells and the homologous CCM. These NPs further caused the photothermal-mediated immunogenic death of tumor cells under NIR radiation, which not only damaged the primary tumor but activated the immune system to enhance the therapeutic efficiency and inhibit tumor metastasis (Supplementary Scheme). Moreover, the CCM corona could be freely adjusted according to the type of tumor, resulting in high therapeutic efficiency in both cell line-derived subcutaneous-xenograft (CDX) models, tongue orthotopic xenograft (TOX) models, immune-competent primary and distant tumor models, and patient-derived xenograft (PDX) models. The modular design of the Au@C-CCM nanoplatforms represents a promising approach for the personalized treatment of various tumors.

## Results

### Preparation and characterization of Au@C-CCM

**Fig. 1:**
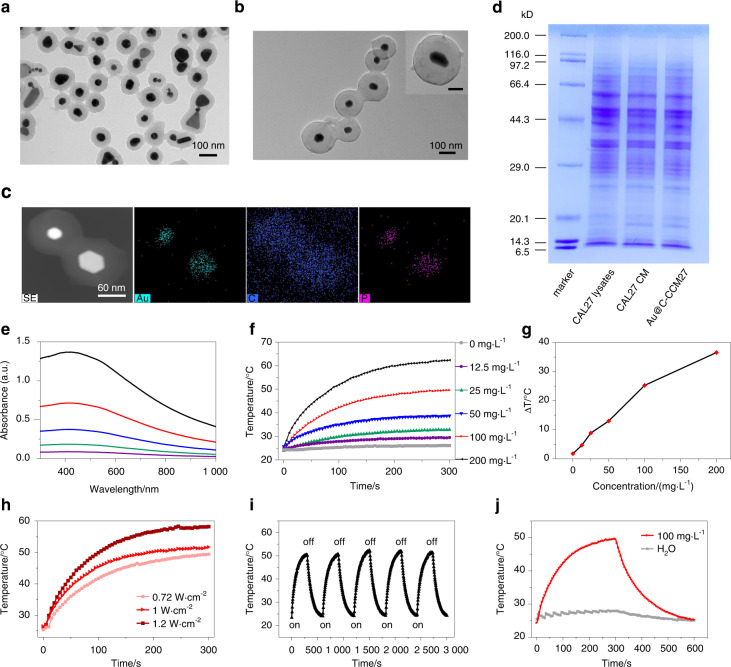
Physicochemical characterization of Au@C and Au@C-CCM.TEM images of **a** Au@C NPs and **b** Au@C-CCM NPs. Inset in the upper right corner: TEM image of a single Au@C-CCM NP, scale bar = 50 nm. **c** EDS elemental mapping of Au@C-CCM. **d** SDS-PAGE and **e** vis-NIR absorption spectroscopy of Au@C-CCM dispersions of the indicated concentrations. **f** Photothermal heating curves of the indicated Au@C-CCM dispersions under 808 nm laser irradiation (1 W·cm^−2^) for 5 min. **g** Plots of temperature change (ΔT) versus the concentration of Au@C-CCM. **h** Temperature change curves of Au@C-CCM dispersion under different laser power densities. **i** Temperature change curve of Au@C-CCM through five on-off laser cycles. **j** Photothermal temperature change curve of 100 mg·L^−1^ Au@C-CCM and water

As to prepare the Au@C-CCM NPs, Au@C NPs were synthesized using perchlorate gold (HAuCl_4_·3H_2_O) as the gold source and glucose (C_6_H_12_O_6_) as the carbon source via a one-step hydrothermal method. After Au@C NPs preparation, the membrane of patient-derived cells (PDC) was extracted via a repeated freeze-thaw process, and then coated on the surface of Au@C NPs via sonication-absorption to form Au@C-CCM. The CAL27 CMs were employed as the model CCM. The morphology of Au@C and Au@C-CCM was investigated by transmission electron microscopy (TEM) (Fig. 1a, b). The spherical Au@C has a designed core-shell structure with a dynamic diameter of approximately 150 nm. The CCM coating did not change the spherical morphology of NPs, which formed a clear organic layer on the surface of Au@C, resulting in a larger size of approximately 250 nm for Au@C-CCM (Fig. S1). The distinct size distribution of these NPs and zeta potential are also presented in Figs. S1, S2. The CCM coating process was investigated by Fourier transform infrared (FTIR) spectroscopy. The spectroscopy of Au@C after CCM coating presented additional peaks of C–H (CH_2_) vibration at 2922 cm^−1^ and P–O vibration at 1061 cm^−1^, which could be attributed to the phospholipids in CCM,^29^ indicating the successful synthesis of Au@C-CCM (Fig. S3). More evidence for the formation of the designed Au@C-CCM was provided by the elemental mapping images of TEM, which showed the characteristic elements of Au, C, and P from the gold core, carbon shell, and CCM corona, respectively (Fig. 1c and Fig. S4). The biochemical structure of CCM before and after coating on Au@C as investigated by Western–Blotting analysis using sodium dodecyl sulfate-polyacrylamide gel electrophoresis (SDS-PAGE) and Coomassie blue staining as well as identification of specific membrane markers. The Au@C-CCM27 showed similar protein banding patterns as the CAL27 CMs and also possessed these specific membrane markers (Fig. 1d and Fig. S5).

Au/carbon nanocomposite materials are well-known for their excellent photothermal performance, which can be reflected by their efficient absorption in the near infrared (NIR) region.^30–33^ Therefore, the absorption of Au@C-CCM was assessed using UV-visible spectroscopy, which showed a broad NIR absorption band as well as a liner relationship between the absorbance at 808 nm and NP concentration (Fig. 1e and Fig. S6a, b). This strong absorbance at 808 nm was expected to induce the desired photothermal conversion effect, which was evaluated by a thermal IR imager as a temperature detector. We also evaluated the photothermal heating curves for Au@C-CCM and water under 808 or 980 nm laser irritation. The maximum temperature for 200 mg·L^−1^ Au@C-CCM for 5 min under 808 nm was 66.8 °C, higher than the maximum temperature under 980 nm. The temperature of water increased by only 1.8 °C after 5 min irradiation by an 808 nm laser, while a 980 nm laser led to a temperature elevation of 17.8 °C (Fig. S6c, d). The photothermal performance of Au@C-CCM showed obvious dependence on the NP concentration, radiation time, and radiation power, which was able to accommodate a 49.6 °C temperature increase under proper conditions (Fig. 1f–h and Fig. S7). Moreover, Au@C-CCM also exhibited significant stability in photothermal performance after five cycles of laser on-off experiments (Fig. 1i), which demonstrates the possibility of multiple PTT sessions during the practical application of tumor treatment. The significant temperature increase could be attributed to the high photothermal conversion efficiency of Au@C-CCM NPs, which was calculated to be 44.2% (Fig. 1j and Fig. S8).

### In vitro cytotoxicity and homologous targeting performance of Au@C-CCM

**Fig. 2:**
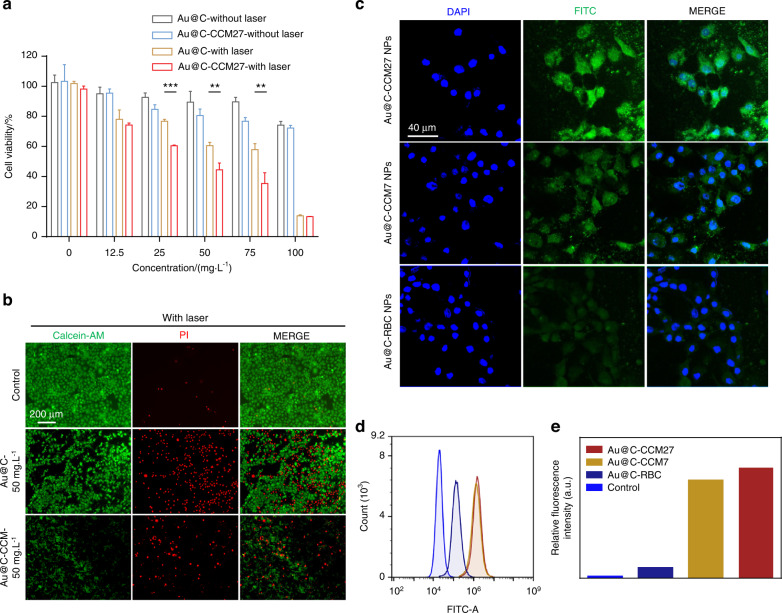
In vitro cytotoxicity assays and homologous targeting performance of Au@C-CCM. **a** Viability of CAL27 cells in the presence/absence of laser irradiation after 24 h incubation with Au@C and Au@C-CCM27 at different concentrations. **b** Calcein-AM and PI staining of CAL27 cells with different treatments. **c** Representative CLSM images of CAL27 cells treated with Au@C-CCM27, Au@C-CCM7, or Au@C-RBC. **d** Flow cytometry and **e** relative fluorescence intensity of CAL27 cells incubated with Au@C-CCM27, Au@C-CCM7, or Au@C-RBC for 4 h. Data are expressed as the mean ± SD (*n* = 3). Statistical significance: **P < 0.01, ***P < 0.001

We next evaluated the cell viability and homologous targeting effect of the Au@C-CCM NPs on the corresponding tumor cells. Herein, three types of Au@C-CCM NPs (i.e., Au@C-CCM27, Au@C-CCM7, and Au@C-CCM6) were prepared through cloaking Au@C with CCMs from three different HNSCC-tumor-derived cell lines (CAL27, SCC7, and HN6). A cell counting kit-8 (CCK-8) assay was used to show the cell viability of CAL27 cells treated with Au@C or Au@C-CCM27 in the presence/absence of laser irradiation (Fig. 2a). In the absence of laser irradiation, both Au@C and Au@C-CCM27 showed negligible deleterious effects at concentrations of 0–100 mg·L^−1^, suggesting good biocompatibility. Upon exposure to an 808 nm NIR laser for 10 min at a power density of 1 W·cm^−2^, we observed enhanced cell death with the increased concentration of both Au@C and Au@C-CCM27. When the NP concentration reached 50 mg·L^−1^, cell viability decreased to 60.58% and 44.41% for the Au@C- and Au@C-CCM27-treated groups, respectively. Au@C-CCM7 and Au@C-CCM6 also manifested similar photothermal toxicity trends with SCC7 cells and HN6 cells, respectively (Fig. S9), indicating the in vitro PTT effect of the Au@C and Au@C-CCM NPs. The pure CCM also did not influence the cell proliferation and cell viability in these HNSCC cells (Fig. S10).

Calcein acetoxymethyl ester (calcein-AM) and propidium iodide (PI) staining assays were conducted to visually distinguish live (green) and dead (red) cells, further demonstrating the antiproliferative effects of Au@C and Au@C-CCM based PTT (Fig. 2b). CAL27 cells mainly remained alive through cocultivation with either Au@C or Au@C-CCM27 at concentrations of 50 mg·L^−1^ without laser (Fig. S11). In contrast, significant cell death was observed in the two groups in the presence of laser irradiation under the same NPs concentration, confirming the photothermal-induced damage to CAL27 cells. A similar photothermal toxicity trend was also observed in HN6 cells and SCC7 cells (Fig. S12).

Through endocytosis experiments, we observed that the green fluorescence signal of Au@C-CCM27-FITC phagocytosed by CAL27 cells increased over time (Fig. S13). Thereafter, we examined the homologous targeting performance of Au@C-CCM in various cell lines to assess personalized customization potential. We prepared three types of biomimetic nanomaterials coated with different CMs from CAL27, SCC7, or mouse red blood cells (RBC) and labeled them with FITC, yielding labeled Au@C-CCM27, Au@C-CCM7, and Au@C-RBC. Then, CAL27 cells were cocultured with these three Au@C-CCM NPs to assess cell uptake as driven by homologous targeting ability. Confocal laser scanning microscopy (CLSM) images indicated significantly increased phagocytosis in the Au@C-CCM27 NPs as compared to Au@C-CCM7 and Au@C-RBC NPs over the observed time period (Fig. 2c). We also used flow cytometry to show that the Au@C-CCM27 group produced the strongest fluorescence intensity compared to the other groups (Fig. 2d, e), thus confirming the homologous targeting ability of Au@C-CCM27 to the corresponding cancer cells.

### In vivo tumor inhibition efficacy of Au@C-CCM in CDX models

**Fig. 3:**
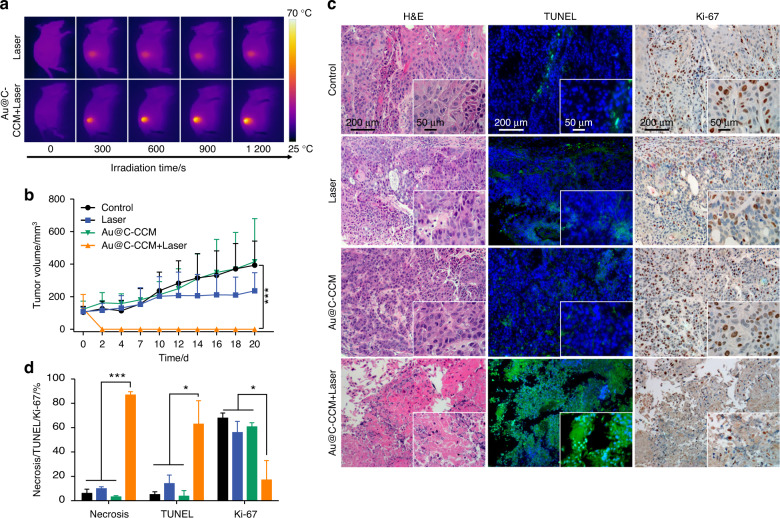
In vivo photothermal anti-cancer performance of Au@C-CCM in CAL27 tumor-bearing CDX models. **a** Thermal images of mice in groups treated with/without Au@C-CCM and 808 nm NIR laser irradiation at various time intervals. **b** Tumor volume change curves following varied treatments (*n* = 3). **c** Histology slices of H&E, TUNEL, and Ki-67 staining of different groups; the corresponding magnification images are shown in the lower right corner. **d** Several randomly selected fields of view under 40× microscope were for quantitative analysis. Data are expressed as the mean ± SD. Statistical significance: *P < 0.05, ***P < 0.001

To evaluate the ability of Au@C-CCM NPs to inhibit tumor growth after PTT in vivo, Au@C-CCM NPs were prepared using the CAL27 cell line and applied to CDX mice bearing CAL27 tumors. Tumor-bearing mice were randomly divided into four groups, including a group with no treatment (control), a group treated with a laser (laser only), a group treated with Au@C-CCM (Au@C-CCM only), and a group treated with both Au@C-CCM and a laser (Au@C-CCM + laser). Mice in the Au@C-CCM only and Au@C-CCM affiliated laser irradiation groups received 6 mg·mL^−1^ Au@C-CCM (200 µL) through intravenous injections. The experiment used an 808 nm laser with a power density of 1 W·cm^−2^, and an IR thermal camera was applied for thermal imaging and recording the temperature changes of the mice upon laser irradiation. We discovered that the temperature of the tumor region increased to 65.8 °C after 20 min of laser irradiation in mice treated with Au@C-CCM, higher than the tumor temperature of mice in the laser group (Fig. 3a and Fig. S14). The results indicated that Au@C-CCM could accumulate into tumors and transfer photo-energy into heat within tumor sites in vivo.

After various treatments, we subsequently recorded body weight and tumor sizes of mice during a 20 day observation. The body weight change curves indicated no statistical loss in body weight among all groups and demonstrate the biosafety of Au@C-CCM and its PTT (Fig. S15). The tumor volume curves (Fig. 3b) revealed that the tumor sizes in the Au@C-CCM-applied laser irradiation group were significantly decreased. In contrast, the untreated mice had a 3.8-fold increase in tumor volume. We further evaluated the therapeutic efficacy via histological staining of tumor tissues, including H&E staining, Ki-67 immunohistochemical (IHC) staining, and triphosphate nick end labeling (TUNEL) immunofluorescent staining (Fig. 3c). For the Au@C-CCM + laser group, the histology of the tumor region indicated significantly increased necrotic lesions, TUNEL-positive cells, and decreased Ki-67 positive cells when compared to the other three groups. The Ki-67 positive percentages were calculated to be 17.0% in the PTT group, 56.04% in the laser group, 60.7% in the Au@C-CCM group, and 67.84% in the untreated group (Fig. 3d). These results suggest that Au@C-CCM based PTT had an inhibitory effect on tumor cell proliferation and was pro-apoptotic.

### Antitumor performance of Au@C-CCM in TOX models

**Fig. 4:**
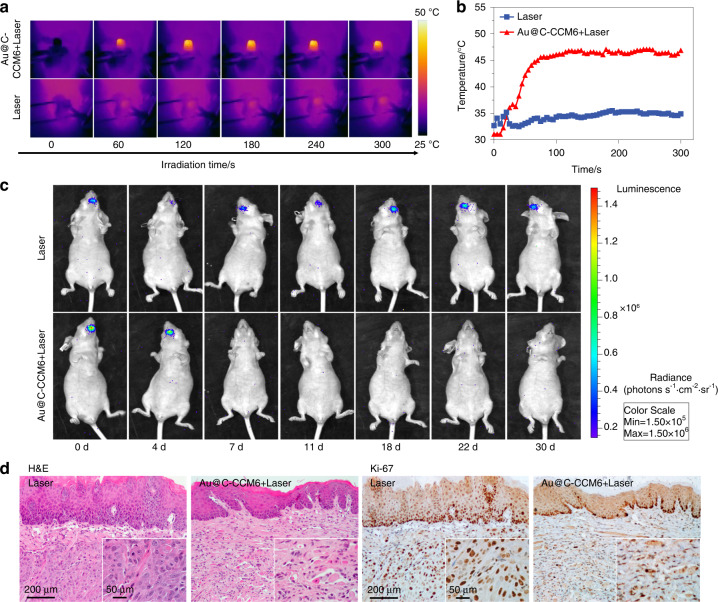
Photothermal anti-cancer performance in orthotopic tongue tumor mouse models. **a** Thermal images of mice after different treatments during various time intervals. **b** Temperature change curve between two groups during defined irradiation times. **c** Representative luminescent images of mice in different groups. **d** Histology slices of H&E and Ki-67 staining of different groups; the representative image of the entire slice is shown in the lower right corner

HNSCC which occurs at oral mucosal sites often influenced by complicated oral microbiota, saliva, gingival crevicular fluid, complement, salivary amylase, and other components, which cannot be simulated by the subcutaneous environment.^34^ Previous studies have shown that orthotopic tumor models can extend the translational relevance of results compared with the subcutaneous milieu.^35,36^ Therefore, we primarily inoculated HNSCC cells (HN6-luciferase cells) submucosally into the left border of the tongue to simulate the tumorigenicity of tongue cancer.^37^ Then, Au@C-CCM6 derived from the CM of HN6-luciferase was prepared and intravenously injected 24 h after the inoculation of tumor cells. The fluorescence signals were acquired, and the tumor areas were laser irradiated with a low power density of 0.72 W·cm^−2^ for 5 min after another 24 h. Through the IR thermal images and temperature curves of the mice, we noted that the maximum temperature in mice that adopted Au@C-CCM6 with laser irradiation increased to 47.1 °C (Fig. 4a, b), a temperature that could induce cell damage.^38^

Tumor signals were present in the tongue of the mice that merely received laser irradiation. While in the mice treated with Au@C-CCM6 plus laser irradiation, fluorescence signals disappeared on the 7th day without relapse for 30 days of observation (Fig. 4c). Moreover, considering the possible difference in the tumor growth pattern between the orthotopic tumor model and the subcutaneous tumor model,^39^ we harvested tongue tissue and stained tumor sections to inspect the tissue morphology. H&E staining demonstrated that tumor cells grew unrestrictedly and disorderly under the mucosa of the tongue in the control mice and mice treated with laser irradiation. In mice treated with Au@C-CCM6 based PTT, the tongue surface returned to a soft texture in gross feature and normal epithelial morphology with laminated cells, and no typical tumor cells could be observed. Ki-67 staining showed that Au@C-CCM6 based PTT could inhibit tumor cells proliferation in tongue tissue (Fig. 4d and Fig. S16). These results indicated that Au@C-CCM could homologously target orthotopic tongue tumor cells yeilding sufficiently accumulate, and induce photothermal ablation of tumor cells under proper conditions, even though the tumors were nonpalpable in the TOX models.

### Evaluation of Au@C-CCM-Mediated PTT induced ICD in vitro and in immune-competent primary and distant tumor models

**Fig. 5:**
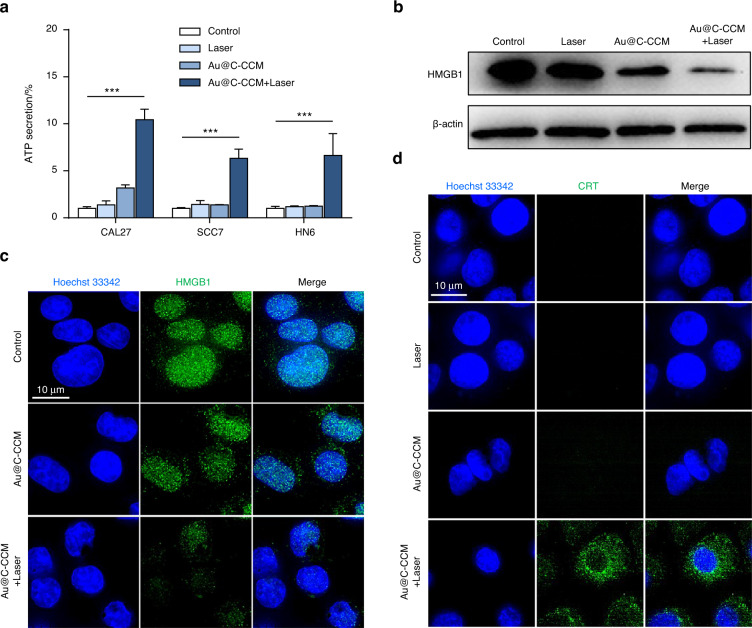
Au@C-CCM-mediated PTT induces immunogenic cell death in vitro. **a** Extracellular ATP levels of CAL27 cells, SCC7 cells, and HN6 cells (*n* = 3). **b** Expression of HMGB1 protein in CAL27 cells after treatment with Au@C-CCM-mediated PTT. **c** Confocal microscopic images of the release of HMGB1 in CAL27 cells after varied treatments. **d** Confocal microscopic images of exposure of CRT in CAL27 cells after varied treatments. Statistical significance: ***P < 0.001

Au@C-CCM-mediated PTT-triggered ICD was evaluated by the detection of ICD markers, including High Mobility Group Box 1 (HMGB1), calreticulin (CRT), and adenosine triphosphate (ATP). Au@C-CCM with 808 nm laser irradiation induced higher ATP secretion in CAL27, SCC7, and HN6 cells as compared to the other groups (Fig. 5a). Western blotting showed the lower expression of HMGB1 in Au@C-CCM-mediated PTT group than the other groups (Fig. 5b). Moreover, CLSM images exhibited the HMGB1-associated green fluorescence dissipated from the nucleus and CRT exposure of CAL27 cells in Au@C-CCM-mediated PTT group (Fig. 5c, d). A similar trend was also displayed in SCC7 cells and HN6 cells (Figs. S17–S20).

**Fig. 6:**
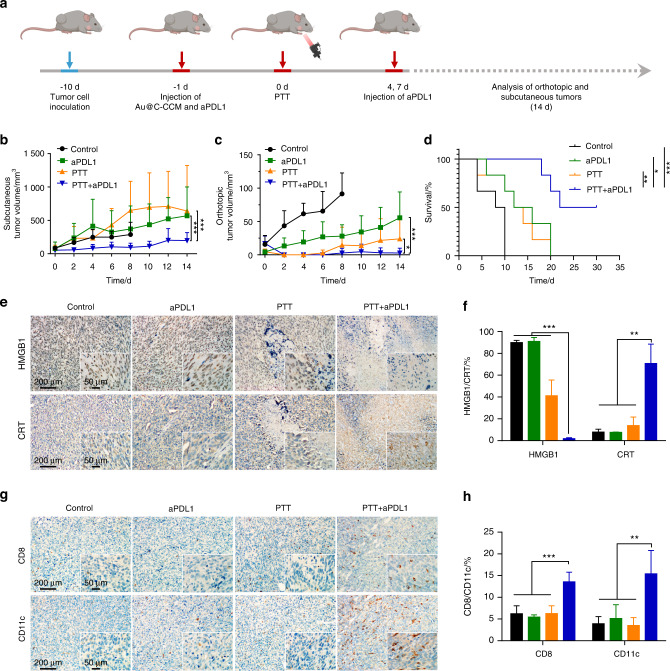
Au@C-CCM-mediated PTT induces immunogenic cell death in vivo. **a** Schematic illustration of the timeline for the in vivo animal study. **b** Subcutaneous tumor volume curves, **c** orthotopic tumor volume curves, and **d** survival rates among the four groups (*n* = 6). **e** HMGB1 and CRT expression and **f** quantified results in different groups. **g** CD8+ and CD11c+ cells expression and **h** quantified results in different groups. The corresponding magnification images are inserted in the lower right corner (**e, g**), and several randomly selected fields of view under 40× microscope were for quantitative analysis (**f**, **h**). Statistical significance: *P < 0.05, **P < 0.01, ***P < 0.001. Schematics were created with BioRender.com

Next, to further verify synergistic Au@C-CCM based PTT and immunotherapy effect in vivo, we inoculated SCC7 cells to the tongue and subcutaneous sites of C3H mouse to simulate primary and distant HNSCC tumors, respectively. Nine days after SCC7 cell inoculation (when the subcutaneous tumor volumes approximately reached ~100 mm^3^), mice were divided into four groups including the control group, a group administered with programmed death-ligand 1 antibody (aPDL1), a group treated with Au@C-CCM7 based PTT, and a group treated with PTT combined aPDL1. Schematic illustration of treatment procedures was depicted in Fig. 6a. The results demonstrated that both the PTT and PTT combined aPDL1 groups exhibited superior photothermal performance (Figs. S21, S22). The subcutaneous tumor volumes of mice in the control group did not demonstrate a rapid growth tendency due to the nutrition intake limitation attributed to the fast-growing orthotopic tongue tumor. PTT combined aPDL1 treatment significantly inhibited both the subcutaneous and the orthotopic tumor growth compared to the other three groups (Fig. 6b, c), illustrating that the tumor inhibition efficacy in the combined treatment group was significantly better than the monotherapy groups. The survival rate of the PTT combined aPDL1 group was the highest among all groups during 30 days of observation (Fig. 6d). Histological staining of tongue tumor tissues demonstrated that PTT combined aPDL1 treatment induced larger areas of necrosis, a significant decrease of Ki-67-positive proliferating cells, and higher cell apoptosis rate than the other groups (Figs. S23, S24). IHC staining of the ICD markers of HMGB1 and CRT in tongue tumors showed that PTT combined aPDL1 treatment led to the significantly decreased expression of HMGB1 and increased CRT exposure compared with the other groups (Fig. 6e, f). We also harvested the subcutaneous tumor and investigated the infiltration of the immune cells into tumor regions. CD8+ (a marker of CTL) and CD11c+ (a marker of DC) cells were significantly increased in the PTT combined aPDL1 group, as compared to the other groups (Fig. 6g, h). These results demonstrated that the release of damage-associated molecular patterns (DAMPs) led to the recruitment of CTLs and DCs in subcutaneous tumors, further promoting antitumor immunity.

### Tumor suppression evaluation of tailored Au@C-CCM in PDX models

PDX models are established by subcutaneously implanting fresh tumor tissues from clinical patients into immune-compromised mice;^40,41^ these models can preserve the histologic and genetic features of donor tumors after implantation.^42^ Therefore, we investigated the customized treatment of homologous Au@C-CCM on PDX models. Two HNSCC patients (defined as 2676 and 2939; clinical information in Table S1) were selected. Optical microscopy images of the diversified cell morphology of their primarily cultured cancer cells were shown in Fig. S25. We prepared two biomimetic nanoplatforms coated with CCMs derived from the patients (named Au@C-2676 and Au@C-2939). We also investigated the cell selectivity in Au@C-2676 to SCC2676, demonstrating that SCC2676 phagocytized more Au@C-2676 NPs, thus exhibiting brighter green fluorescence than cells incubated with Au@C-2939 NPs (Fig. S26). Au@C-2676 and Au@C-2939 were then administered to the PDX model established from the patient defined as 2676 at a dose of 6 mg·mL^−1^ (200 μL) via the tail vein injection, and various treatments similar to those in the CDX models were performed.

**Fig. 7:**
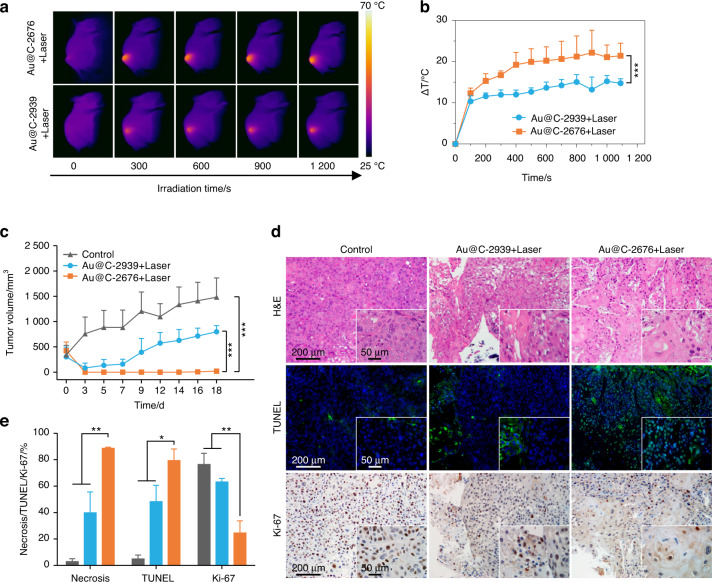
Photothermal anti-cancer performance in HNSCC PDX models. **a** Thermal images of mice and **b** temperature change curve of tumors in PDX models (number: 2676) under laser irradiation after intravenous treatment with Au@C-2676 and Au@C-2939 (*n* = 3). **c** Tumor volume curves after PTT between different groups during the same observation time intervals (*n* = 3). **d** Histology slices of H&E, Ki-67, and TUNEL staining of different groups; The corresponding magnification images are inserted in the lower right corner. **e** Several randomly selected fields of view under 40× microscope were for quantitative analysis. Statistical significance: *P < 0.05, **P < 0.01, ***P < 0.001

As shown in the IR thermal images, the tumor region in the PDX model that received Au@C-2676 was brighter than that in mice which received Au@C-2939, demonstrating higher PTT efficacy of Au@C-2676 (Fig. 7a). We further compared the overall temperature change between the mice that received different treatments (Au@C-2676 and Au@C-2939). The temperature change of the Au@C-2676 group was significantly higher than that of the Au@C-2939 group (Fig. 7b). The constant temperature plots of the tumor region with Au@C-2676 revealed that irradiation caused the temperature to promptly increase by over 10 °C within 1 min and exceed 50 °C within 75 s. In contrast, the tumor region with Au@C-2939 took more than 18 min to reach 50 °C with an identical laser power density (Fig. S27).

Moreover, to test the treatment efficiency of customized Au@C-CCM in PDX models, we measured the tumor volume and body weight of mice among Au@C-2939 with laser group, Au@C-2676 with laser group and the control group for 18 days. The tumor volume curves revealed that tumor growth in the Au@C-2676 group with PTT were significantly inhibited compared to other groups. The control mice had a 4.4-fold increase in tumor volumes. A 2.6-fold increase was observed in the Au@C-2939 group (Fig. 7c). There was no statistical loss in body weight of Au@C-2676 based PTT group. While the body weight of mice in the control group decreased distinctly, probably due to the high tumor malignancy (Fig. S28). These results illustrated that Au@C-2676 resulted in a much more efficient treatment than Au@C-2939, which most likely results from the sufficient accumulation of the homologous NPs within tumors.

For the group treated with Au@C-2676 plus the laser, H&E staining, TUNEL staining, and Ki-67 staining results also demonstrated significant necrosis, cell apoptosis, and cell proliferation-inhibition in Au@C-2676 with laser group compared to the other groups. We also found that Au@C-2676 with laser led to more thorough tumor cells degeneration and more significant interstitial fibrous tissue hyperplasia accompanied by hyaline degeneration than the Au@C-2939 with laser and the control group (Fig. 7d, e).

### In vivo biosafety of Au@C-CCM

The biosafety issues of nanomedicines are paramount for their bio-application and clinical translation.^43^ Au@C-CCM27 was also intravenously injected into healthy mice, and subsequently blood samples were collected and analyzed at different time points. Main organs samples were also collected for histology analysis. Au@C-CCM27 treatment did not produce abnormal indicators of blood biochemistry, including alanine aminotransferase (ALT), aspartate transaminase (AST), blood urea nitrogen (BUN), creatinine (CRE), or uric acid (UA) (Fig. S29). Additionally, H&E staining showed no observable damage to main organs, including the heart, liver, spleen, lung, and kidney (Fig. S30). In order to further evaluate the biosafety of this biomimetic nanoplatform, we prepared CCM vesicles using HNSCC cells and labeled them with FITC to track their metabolic fate in vivo. We acquired fluorescent signals at various time intervals and discovered that the signals decreased and finally disappeared on the 11th day posttreatment (Fig. S31), indicating the elimination of CCM vesicles. These results demonstrated the safety of the biomimetic nanoplatform in vivo to some extent.

Given PDC-CM-camouflaged nanotechnology and customized PTT (PCMPT) is tailored for HNSCC patients, PCMPT could be applied as a promising therapeutic strategy for early stage HNSCC via biopsy sampling. Through HNSCC cells primary culture from biopsy samples, the CMs extraction and cloaking Au@C, the personalized biomimetic NPs based PTT could be administered to patients, and performed as a pre-operative therapy that will effectively control tumor progression or even ablate tumor tissues completely, allowing for reducing the risk of HNSCC development.

## Discussion

The emergence of biomimetic nanotechnology provides a significant foundation for research in tumor therapies. Modular design is a methodology that satisfies different market demands through the selection and combination of self-contained functional units.^44^ In our research, modular design was adopted to customize a series of biomimetic nanomaterials coated with CMs from HNSCC commercial cell lines or PDC and study their therapeutic effects on matched cell-lines or PDX models. Au@C-CCM NPs were constructed from various CCMs, including three HNSCC cell lines and two PDCs. Our results indicate that different CM-wrapped Au@C-CCM NPs possessed homologous targeting properties and their mediated PTT had superior effects in matched cell-line derived models and paired PDX models, even in occult tumor focus. In addition, Au@C-CCM NPs mediated PTT directly caused tumor cell death and further induced ICD effects to cause a cascade reaction, including an increase in the infiltration of CTLs, thus creating an immune-responsive tumor microenvironment and significantly improving the efficiency of aPDL1.

A variant of PTAs have been constructed for PTT, including metal nanostructures, carbon-based nanomaterials, organic dyes and polymers, and semiconductor nanomaterials. Au nanomaterials have become one type of the most widely used PTAs due to their low toxicity, good biocompatibility, chemical inertness, and tunable LSPR effect.^45^ Specifically, due to optical resonance peak manifests in the NIR region, Au nanomaterials have superior clinical application potential with PTT.^46^

Although multiple advanced biomimetic nanomaterials have been constructed, most of these reported works had their own limitations, such as supporting only passive accumulation, low circulation time, increasing immunogenicity, and targeting to normal cells.^47^ Au@C-CCM derived from patient cancer cells maintains the tumor-specific binding proteins, facilitating the homologous targeting of CCM-coated NPs to homotypic cancer cells. Moreover, the modular design of Au@C-CCM and preclinical models was constructed pioneeringly, supporting the design and application of personalized biomaterials in different scenarios, which would promote the clinical translation of biomimetic nanomaterials. Obviously, there is a limited supply of CCM from resected tumors or biopsies as opposed to cultured cell lines in this study, and it is also necessary to confirm that all carcinogenic potential are completely eliminated. Moreover, the reasonable mechanism and clinical application are need to be further investigated.

While Au@C-CCM supported an efficient PTT effect for HNSCC treatment, it should be possible to improve the photothermal conversion efficiency of the Au core, for example, by tuning its size and/or morphology.^48^ It should also be possible to employ alternative PTT agents and/or to deploy combinations of Au nanomaterials with other agents.^49^ In addition, the increasing of targeting efficiency would be achieved by modification of the Au NPs surface, such as conjugating PEGylated Au NPs with specific membrane antigen-antibody, to improve drug-loading capacity.^50^ Modification of CCMs on the NPs by lipid insertion, membrane fusion, genetic engineering and metabolic engineering could also with enhanced targeted functionality towards tumors.^51^

Several molecular mechanisms have been reported that deepen understanding of homologous targeting as mediated by CCM. Surface proteins with homophilic adhesion domains, such as Thomsen-Friedenreich, E-cadherin, and epithelial cell adhesion molecules, are responsible for the multicellular aggregate formation, which can be reserved in Au@C-CCM.^52^ In our future research, we plan to sequence the tumor tissues of HNSCC patients, screen and investigate differentially expressed adhesion-related molecules or proteins that participate in homologous targeting, and stratify patients according to the expression level of these biomarkers. We believe this will allow us to predict the efficacy of Au@C-CCM mediated PTT (CCM originated from their own cancer cells), and design individualized therapies suitable for each patient based on these biomarkers.

In summary, a series of Au@C-CCM NPs and modular models were constructed to evaluate the translational potential for the individualized treatment for HNSCC. The nanoplatform retained its effective photothermal properties and also contributed to the customized treatment of HNSCC. In the cell experiments, Au@C-CCM demonstrated homologous targeting and photothermal ablation of the donor cells in vitro. After intravenous injection in vivo, Au@C-CCM was able to accumulate in tumors and mediate tumor inhibitions upon laser irradiation in HNSCC CDX, TOX, and PDX models. Although much more work needs to be done for systematic investigation of biomimetic nanoplatforms, this study reinforces their feasibility for customized treatment of HNSCC and promotes translation.

## Materials and methods

### Synthesis of Au@C core-shell NPs

A total of 30 mL of ddH_2_O was added to 900 mg of glucose. After constant stirring for 30 min, 5.9 mg of HAuCl_4_·3H_2_O was added, with additional stirring for another 30 min. The whole solution was then transferred to a reactor at 180 °C for 3 h.

### Preparation of CCM and Au@C-CCM NPs

The cells were collected and suspended in 1 mL of 0.25× PBS buffer consisting of one EDTA-free miniprotease inhibitor tablet (complete™, Mini, EDTA-free protease inhibitor cocktail, Roche), 20 mmol·L^−1^ Tris-HCl, 10 mmol·L^−1^ KCl, and 2 mmol·L^−1^ MgCl_2_ per 10 mL.^25^ The cells were then subjected to a freeze-thaw process (−80 °C to room temperature, three times) followed by sonication (37 kHz) for 1 min.^53,54^ Sediment was collected by centrifugation at 14 000 r·min^−1^ for 15 min at 4 °C three times, dispersed in 1× PBS and centrifuged (3 000 r·min^−1^, 4 °C) for 30 min. The supernatant containing CCM was obtained.

The mixture containing CCM and Au@C was sonicated (4 °C) for 10–14 s to obtain Au@C-CCM NPs, and resuspended in 1 mL 1× PBS for use.

### Characterization of CCM proteins

SDS-PAGE was used for characterizing the CCM proteins in CAL27 lysates, CCM vesicles, and Au@C-CCM. After quantification with a BCA assay kit (Beyotime, China) and heat denaturation, samples with equal protein quantities were loaded onto a gel (GenScript, China) in a Mini Trans–Blot cell system (BIO-RAD, CA, USA) based on the manufacturer’s instructions. Coomassie Blue was used for protein staining. Membrane markers of Na-K-ATPase (1:1 000, 3031 S, CST) and EGFR (1:5 000, ab32562, Abcam) were also detected through western blotting.

### Cell cytotoxicity assay

CAL27, HN6, and SCC7 cells were seeded in 96-well (1 × 10^4^ cells per well) plates for incubation, and then in a medium containing NPs (0–100 mg·L^−1^) for 24 h. Plates were detected using a microplate reader (Gene5 BioTek, USA) 2 h after adding 10% CCK-8 (Beyotime, China).

### Photothermal performance in vitro

For Au@C-CCM NPs, dispersions of various concentrations (0–200 mg·L^−1^) were irradiated with an 808 nm NIR laser for 10 min at 1 W·cm^−2^. Thermal images and temperature changes were recorded using a thermo imaging camera (FOTRIC 226 S, China).

In cell experiments, CAL27 cells were seeded in 96-well (1 × 10^4^ cells per well) plates for incubation, and then in a medium containing NPs (0–100 mg·L^−1^) for 24 h. Cells were then irradiated with an 808 nm laser at 1 W·cm^−2^ for 10 min. Plates were detected using a microplate reader (Gene5 BioTek, USA) 2 h after adding 10% CCK-8 (Beyotime, China). For calcein-AM/PI staining, CAL27 cells were incubated with Au@C or Au@C-CCM NPs (50 mg·L^−1^) for 24 h and irradiated for 10 min at 1 W·cm^−2^. After staining with calcein-AM and PI (L3224, Thermal Scientific) solution for 20–30 min, a biological inverted microscope (Zeiss, Germany) was used to observe live and dead cells. Similar methods were also applied to HN6 and SCC7 cells.

### Endocytosis and homologous targeting experiment

CAL27 cells were cocultured with Au@C-CCM27 NPs (25 mg·L^−1^, labeled FITC) for 1 h, 2 h, 4 h, and 8 h for the endocytosis experiment. CAL27 cells were incubated with 25 mg·L^−1^ varied NPs (Au@C-CCM27-FITC, Au@C-CCM7-FITC, and Au@C-RBC-FITC) to test the homologous targeting properties of Au@C-CCM. Image acquisition was acquired on a confocal microscope (Zeiss, LSM 800).

For flow cytometric analysis, CAL27 cells were incubated with Au@C-CCM27 NPs, Au@C-CCM7 NPs, and Au@C-RBC NPs for 4 h and then with pure DMEM for 1 h.^53,55^ Data were collected using NovoCyte (ACEA, USA). Analysis was performed using NovoExpress software.

### ATP release assay

After treatment with Au@C-CCM (50 mg·L^−1^) and laser irradiation (1 W·cm^−2^, 10 min per well), the supernatant of CAL27, SCC7, and HN6 cells was analyzed with an enhanced ATP assay kit (Beyotime, S0027) and detected with a luminometer (Tecan Spark, Switzerland) according to the manufacturer’s protocol.

### Western blotting analysis and immunofluorescence staining of HMGB1

CAL27 cells (2 × 10^5^ cells per well) were seeded in a 12-well plate and were treated with 50 mg·L^−1^ Au@C-CCM for 24 h. 6 h after PTT, the whole protein was quantified, separated, and transferred onto polyvinylidene difluoride (PVDF) membranes (Millipore, ISEQ00010).^56^ After blocking with 5% milk, PVDF membranes were incubated with primary antibody HMGB1 (1:1 000, ab227168, Abcam) and secondary antibody. Detection of proteins was performed on a gel Imaging System (Tanon, China).

For immunofluorescence staining, CAL27 cells (8 × 10^4^) were cultured in a glass-bottom dish and then 50 mg·L^−1^ Au@C-CCM was added and exposed to 10 min of irradiation. After primary antibody and secondary antibody (Alexa Fluor 488-conjugated, 1:500, 4340 S, CST) incubation, cells were stained with Hoechst 33342, and images were obtained (GE DeltaVision OMX SR). Similar methods were also applied to HN6 and SCC7 cells.

### Immunofluorescence staining of CRT

CAL27 cells (8 × 10^4^ cells per dish) were treated with 50 mg·L^−1^ Au@C-CCM for 24 h. After 10 min of irradiation, cells were incubated with CRT (1:200, 12238 S, CST) and secondary antibodies. Images were obtained with a GE DeltaVision OMX SR and a confocal microscope. Similar methods were also applied to HN6 and SCC7 cells.

### Primary cell culture of PDC

PDC cell culture was performed referred to our previous study.^57^ Briefly, specimens were digested in a mixture containing collagenase type IV, liberase, and deoxyribonuclease type I. Consistent with previous reports, a complete F medium was used for further culture.^58,59^

### Animal models

CAL27 cells (1 × 10^6^ cells per mouse) were injected into BALB/c nude mice (female, 6 weeks old) subcutaneously.^60^ HN6-Luc cells (1 × 10^5^ cells in 25 μL 1× PBS) were injected submucosally into the left border of the BALB/c nude mouse tongue.^37^ SCC7 cells (1 × 10^5^ cells in 25 μL 1× PBS) were identically injected into the tongues of C3H mice (female, 5 weeks old) and inoculated (1 × 10^6^ cells in 100 μL 1× PBS) subcutaneously.

PDX models were established according to our previous report.^61^ Briefly, each fresh tumor fragment from an HNSCC patient numbered SCC2676 was subcutaneously engrafted into BALB/c nude mice. After vigorous tumor growth, the mice were tested with further treatment.

### In vivo PTT experiment and synergistic PTT and immunotherapy

Mice were randomly assigned to four groups when subcutaneous tumors reached approximately 100 mm^3^: control, laser, Au@C-CCM, Au@C-CCM + laser. PTT condition: Au@C-CCM dispersions (200 µL, 6 mg·mL^−1^, intravenously) with irradiation at 1 W·cm^−2^ for 20 min. For the TOX tumor models, the condition was 0.72 W·cm^−2^ for 5 min to avoid tissue damage. For immune-competent primary and distant tumor models, the groups were as follows: control, aPDL1, PTT, PTT + aPDL1. For PTT treatment, mice were administered with Au@C-CCM (6 mg·mL^−1^, 200 μL, intravenously). 24 h later, mice tongue tumors were irradiated with an 808 nm laser (1 W·cm^−2^) for 5 min, while subcutaneous tumors were left untreated. For aPDL1 treatment, mice were injected with aPDL1 (0.012 5 mg per mouse by intraperitoneal injection^62^) three times (before PTT, and then 4 and 7 days after PTT.^63,64^) For PDX models, the groups were as follows: control, Au@C-2939 + laser, Au@C-2676 + laser. The PTT condition was identical to the subcutaneous models. Subcutaneous tumor volume was calculated according to the formula: 0.5 × length × width^2^.^65^ Orthotopic tongue tumor volume was calculated according to: AB^2^(π/6), where A is the longest dimension and B is the dimension perpendicular to A.^66^

### Paraffin sectioning and staining

Tumors were collected and embedded in paraffin and sectioned into 5 µm slices using a microtome (Leica, Germany). The SCC7 subcutaneous tumors were harvested 3 days after varied treatments and stained with CD8 (1:400, GB13429, Servicebio Technology) and CD11c (1:200, GB11059, Servicebio Technology). H&E, TUNEL (Servicebio Technology), Ki-67 (1:300, GB111499, Servicebio Technology), CRT (1:100), and HMGB1 (1:400) antibodies were also applied. Image acquisition was obtained with a Zeiss AXIO Vert A1 inverted fluorescence microscope, and calculations were performed using ImageJ.

### In vivo biosafety examination

Main organs (heart, liver, spleen, lung, and kidney) from mice were H&E stained to observe morphology after Au@C-CCM administration (200 µL, 6 mg·mL^−1^), and blood samples were acquired for biochemistry analysis. CAL27 CM vesicles (200 µL, 1 mg·mL^−1^) were prepared (Miniextruder, Avanti) and intravenously injected into healthy mice. Fluorescence was acquired using an IVIS SpectrumBL (PerkinElmer).

### Ethics

Tumor tissues were collected from Shanghai Ninth People’s Hospital of the Shanghai Jiao Tong University School of Medicine and complied with the Declaration of Helsinki and was approved by the Ethics Committee of Shanghai Ninth People’s Hospital. Written informed consent was obtained from all study participants prior to the study. All animal experiments were performed in accordance with the guidelines of the Laboratory Animal Ethics Committee in Ninth People’s Hospital, Affiliated with Shanghai Jiao Tong University School of Medicine.

### Statistical analysis

Data are presented as mean ± standard deviation (SD). Two-tailed *t*-test or two-way analysis of variance (ANOVA) was performed depending on the number of treatment groups and distribution. Statistical analyses were performed using Graphpad Prism version 8. A *P*-value <0.05 was considered statistically significant.

## Supplementary information

Supplementary Information

Supplementary Scheme

## Data Availability

All data associated with this study are presented in the paper or the Supplementary Information. Any other relevant data are available from the corresponding author upon reasonable request.
