# Supplementary material for: A biomimetic nanoplatform for customized photothermal therapy of HNSCC evaluated on patient-derived xenograft models
Source: Int J Oral Sci. 2023 Feb 10;15:9. doi: 10.1038/s41368-022-00211-2 (PMC9918549; doi:10.1038/s41368-022-00211-2)
Supplement: Supplementary file 1 — Supplementary Information [file 41368_2022_211_MOESM1_ESM.docx]

Supplementary Information

**A Biomimetic Nanoplatform for Customized Photothermal Therapy of HNSCC Evaluated on Patient-Derived Xenograft Models**

**Running title:** Modular designed evaluation of Au@C-CCM

*Qi Wu^1^, Lan Chen^1^, Xiaojuan Huang**^1,^*, Jiayi Lin^2^, Jiamin Gao^1^, Guizhu Yang^1^, Yaping Wu^1^, Chong Wang^1^, Xindan Kang^1^, Yanli Yao^1^, Yujue Wang^1^, Mengzhu Xue^1^, Xin Luan^2^, Xin Chen^3^, Zhiyuan Zhang^1,^*, and Shuyang Sun^1,^**

^1^Department of Oral and Maxillofacial-Head & Neck Oncology, Shanghai Ninth People’s Hospital, Shanghai Jiao Tong University School of Medicine; College of Stomatology, Shanghai Jiao Tong University; National Center for Stomatology; National Clinical Research Center for Oral Diseases; Shanghai Key Laboratory of Stomatology; Shanghai Research Institute of Stomatology, Shanghai 200011, China

^2^Shanghai Frontiers Science Center for Chinese Medicine Chemical Biology, Institute of Interdisciplinary Integrative Medicine Research, Shanghai University of Traditional Chinese Medicine, Shanghai 201203, China

^3^School of Chemical Engineering and Technology, Shaanxi Key Laboratory of Energy Chemical Process Intensification, Institute of Polymer Science in Chemical Engineering, Xi’an Jiao Tong University, Xi’an, Shaanxi 710049, China

The official email addresses of all authors:

Qi Wu (wuqi19510@sjtu.edu.cn); Lan Chen (lanchen@sjtu.edu.cn); Xiaojuan Huang (184466@sjtu.edu.cn); Jiayi Lin (linjiayilumos@163.com); Jiamin Gao (gaojiamin18916212273@sjtu.edu.cn); Guizhu Yang (yangguizhu@sh9hospital.org.cn); Yaping Wu (wuyaping@sjtu.edu.cn); Chong Wang (wangchongkq@sjtu.edu.cn); Xindan Kang (kxindan@sjtu.edu.cn); Yanli Yao (yaoyanli@shsmu.edu.cn); Yujue Wang (wyjsjtu@sjtu.edu.cn); Mengzhu Xue (xuemz@sjtu.edu.cn); Xin Luan (luanxin@shutcm.edu.cn); Xin Chen (chenx2015@xjtu.edu.cn); Zhiyuan Zhang (zhzhy@sjtu.edu.cn); Shuyang Sun (sunshuyang@sjtu.edu.cn)

Correspondence:

Shuyang Sun, M.D., Ph.D.

Chief Scientist of The National Key Research and Development Program of China, Department of Oral and Maxillofacial-Head Neck Oncology, Shanghai Ninth People’s Hospital, College of Stomatology, Shanghai Jiao Tong University School of Medicine

E-mail: sunshuyang@sjtu.edu.cn

Tel: +86-21-23271699; Fax: +86-2163136856

Zhiyuan Zhang, M.D., Ph.D.

Distinguished Professor, Shanghai Jiao Tong University, Academician of Chinese Academy of Engineering, Department of Oral and Maxillofacial-Head Neck Oncology, Shanghai Ninth People’s Hospital, College of Stomatology, Shanghai Jiao Tong University School of Medicine

E-mail: zhzhy@sjtu.edu.cn

Tel: +86-21-23271699; Fax: +86-2163136856

Xiaojuan Huang, Ph.D.

Department of Oral and Maxillofacial-Head Neck Oncology, Shanghai Ninth People’s Hospital, College of Stomatology, Shanghai Jiao Tong University School of Medicine

E-mail: 184466@sjtu.edu.cn

Tel: +86-21-23271699; Fax: +86-2163136856

These authors contributed equally: Qi Wu, Lan Chen


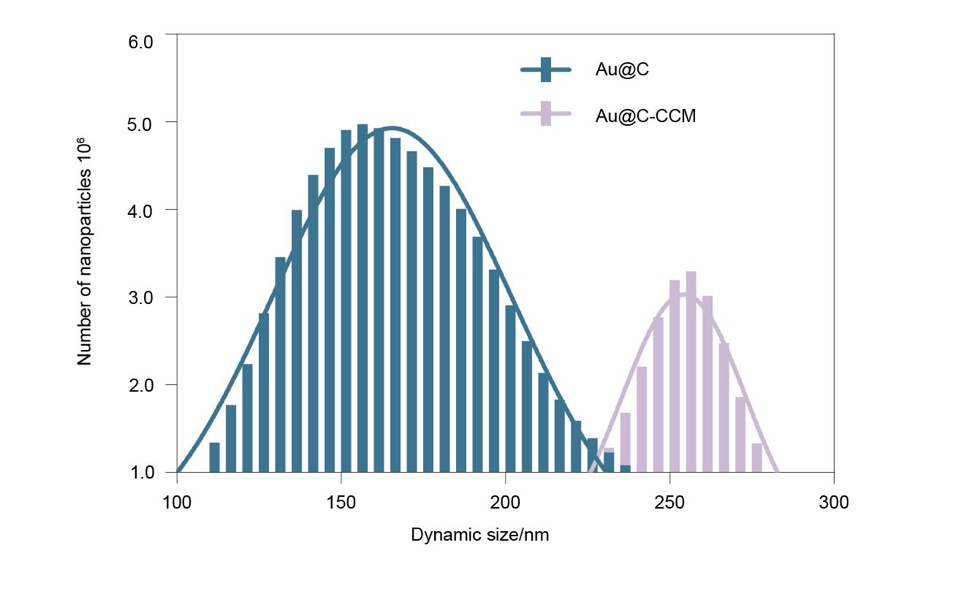


**Fig. S1** Dynamic diameters of Au@C and Au@C-CCM.


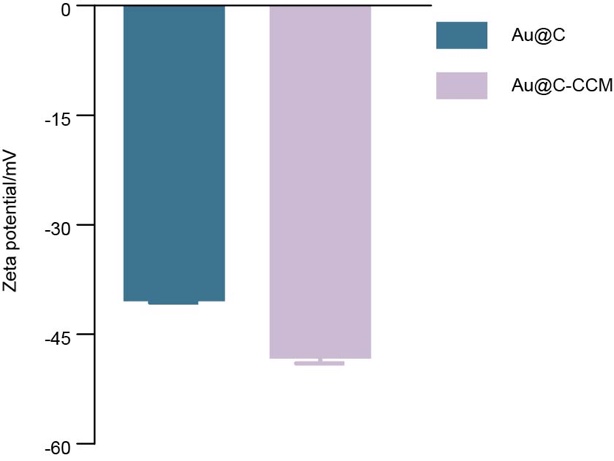


**Fig. S2** Zeta potential of Au@C and Au@C-CCM.


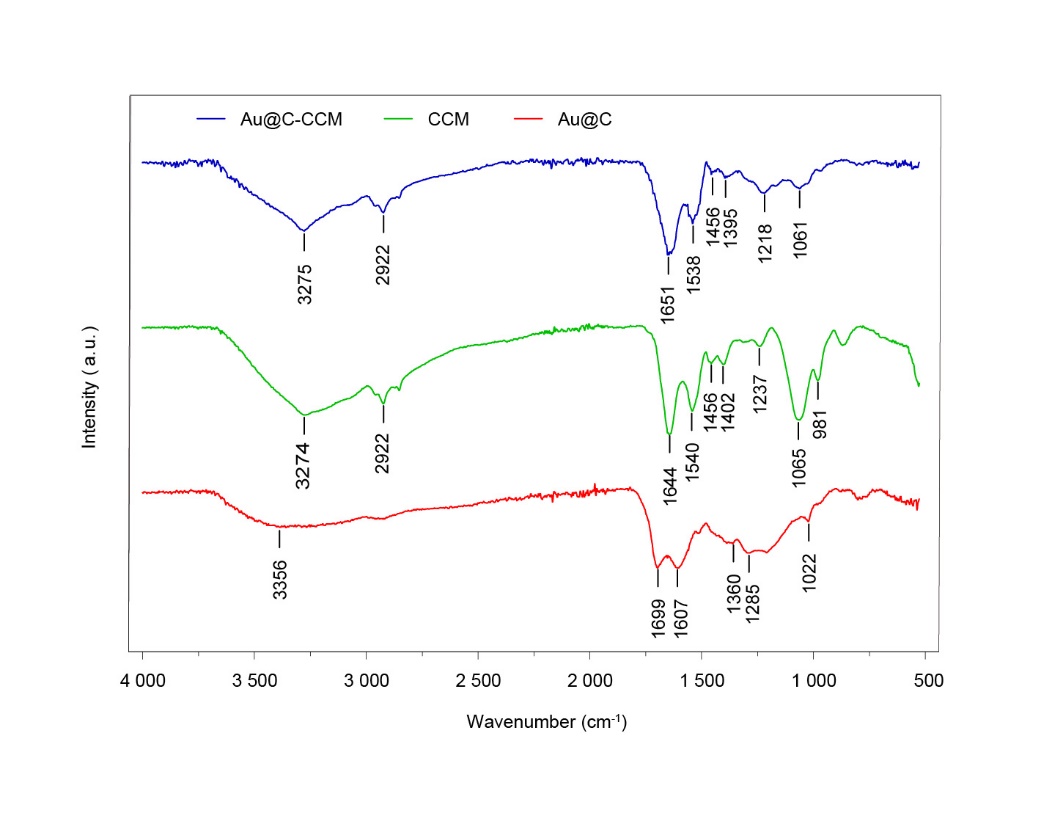


**Fig. S3** FTIR spectroscopy of Au@C, CCM, and Au@C-CCM.


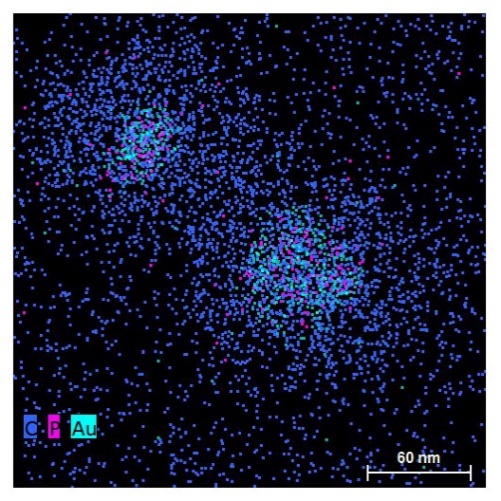


**Fig. S4** EDS elemental mapping with merged carbon, phosphorus and gold window of Au@C-CCM nanosystem.


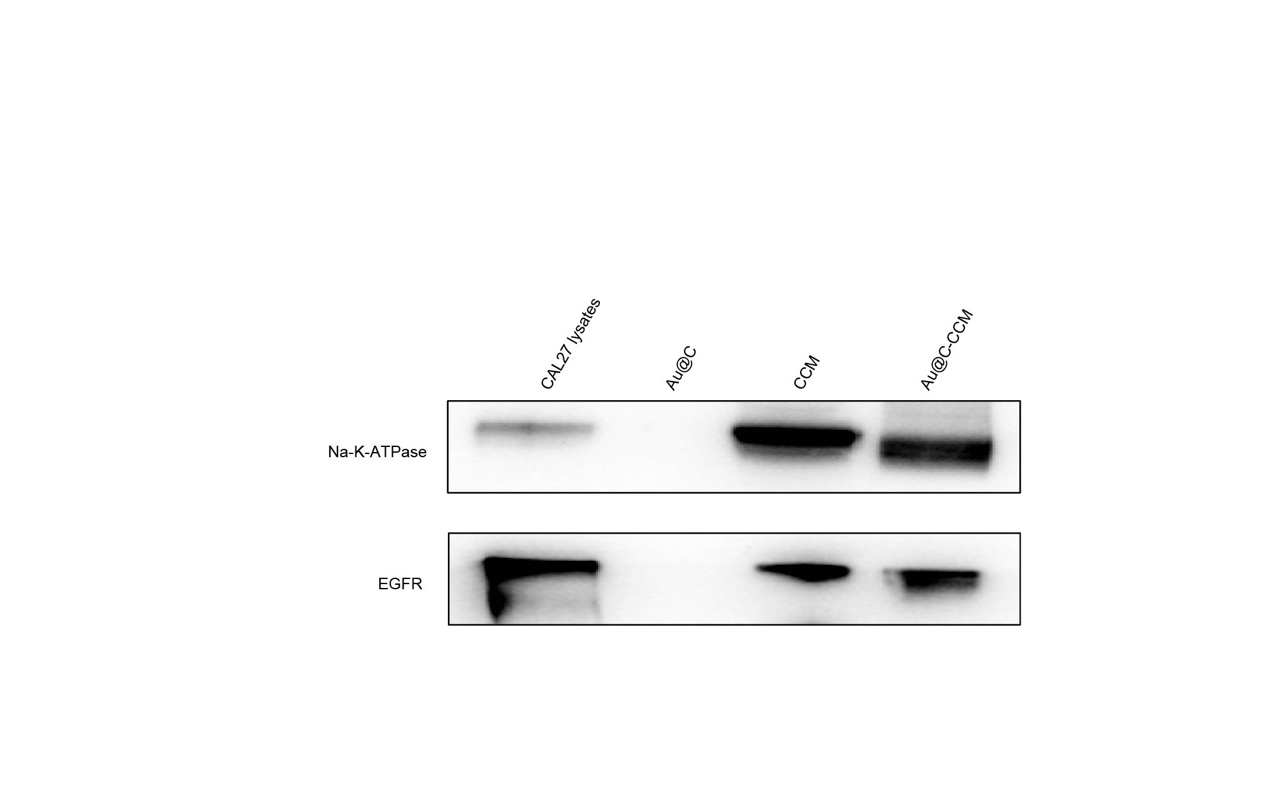


**Fig. S5** Western blotting analysis of membrane-specific protein markers.


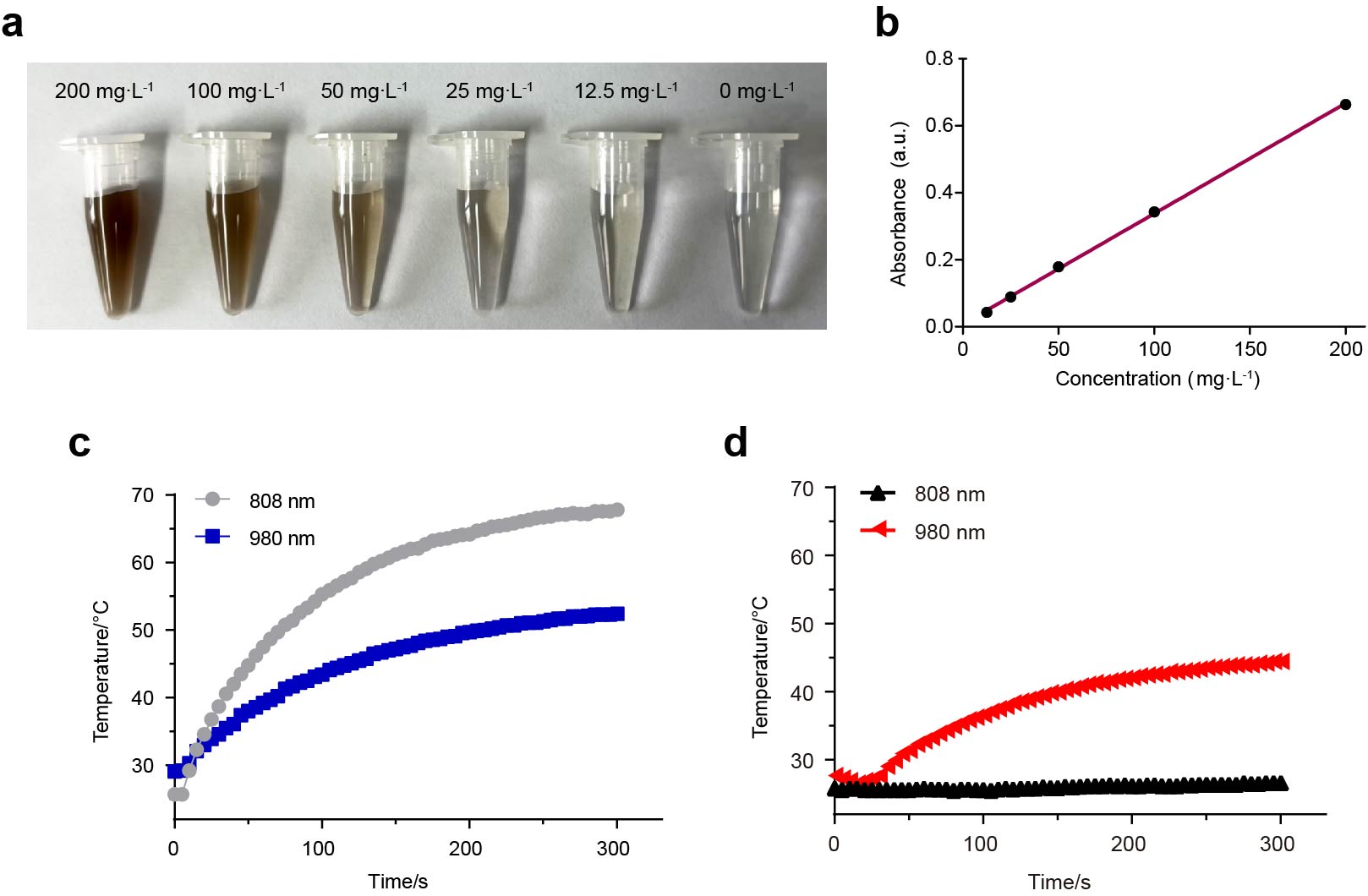


**Fig. S6** Temperature change comparison of Au@C-CCM dispersion and water between 808 nm and 980 nm laser irradiation. **a** Photographs of [aqueous](javascript:;) [solution](javascript:;)s with Au@C-CCM in different concentrations (0, 12.5, 25, 50, 100, 200 mg·L^-1^). **b** Concentration dependent absorbance of Au@C-CCM at 808 nm. Photothermal heating curves of **c** 200 mg·L^-1^ Au@C-CCM dispersions and **d** water under 808 or 980 nm laser irradiation (1 W·cm^-2^) for 5 min.


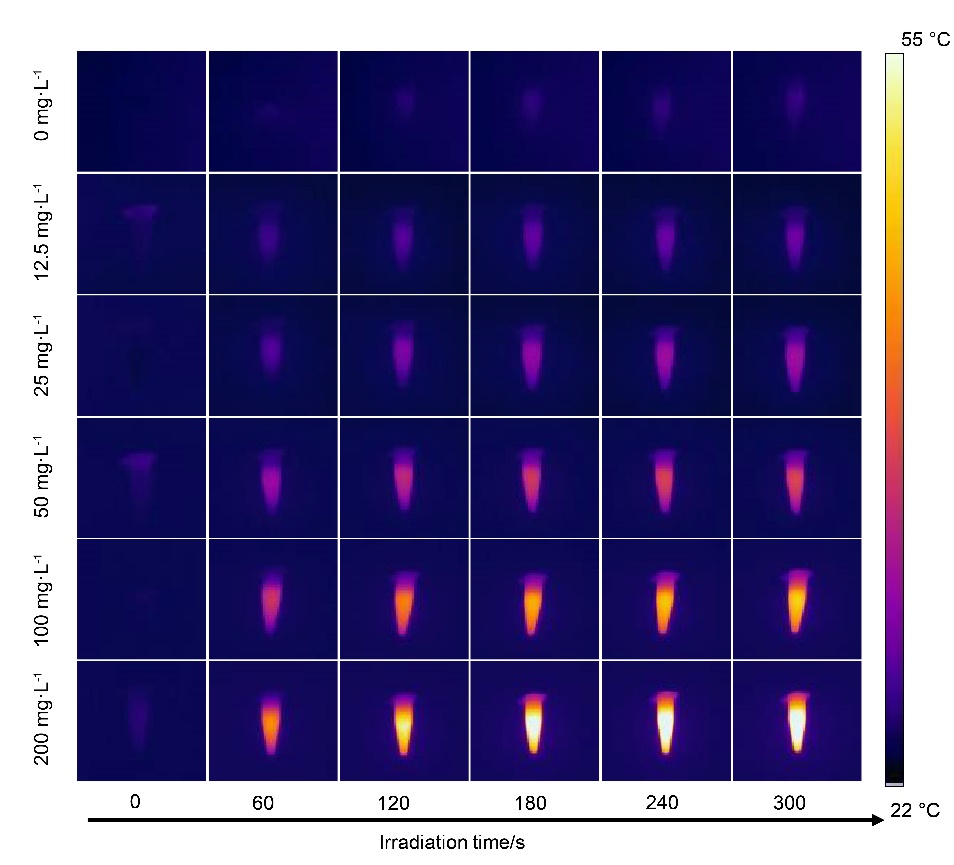


**Fig. S7** Thermal images of aqueous solutions containing Au@C-CCM in different concentrations after 808 nm laser irradiation.


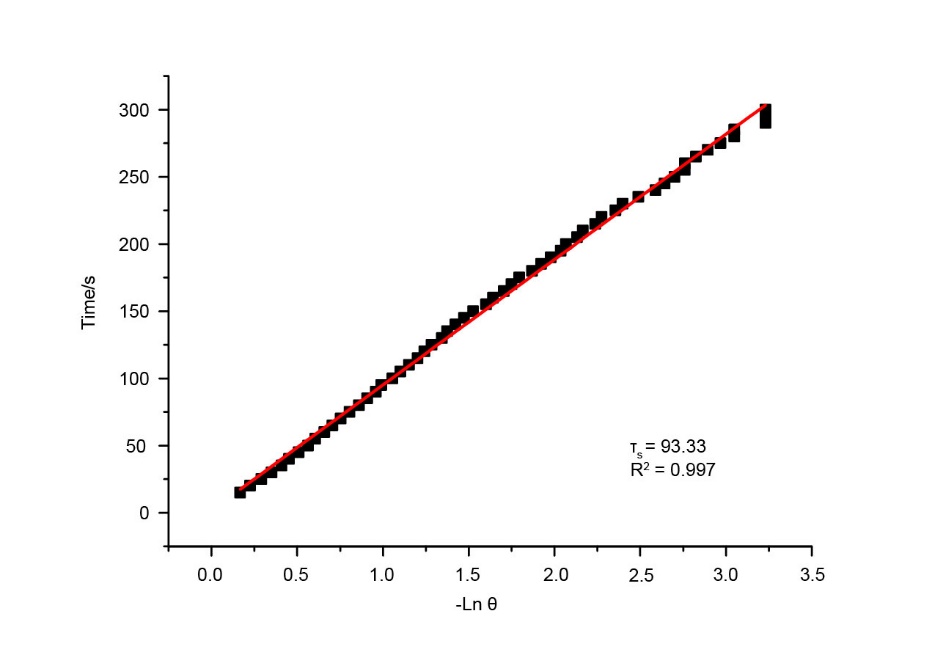


**Fig. S8** Time constant for heat transfer from the system (*τ_s_*), calculated from the cooling period of panel (Fig. 1j).^1, 2^

**
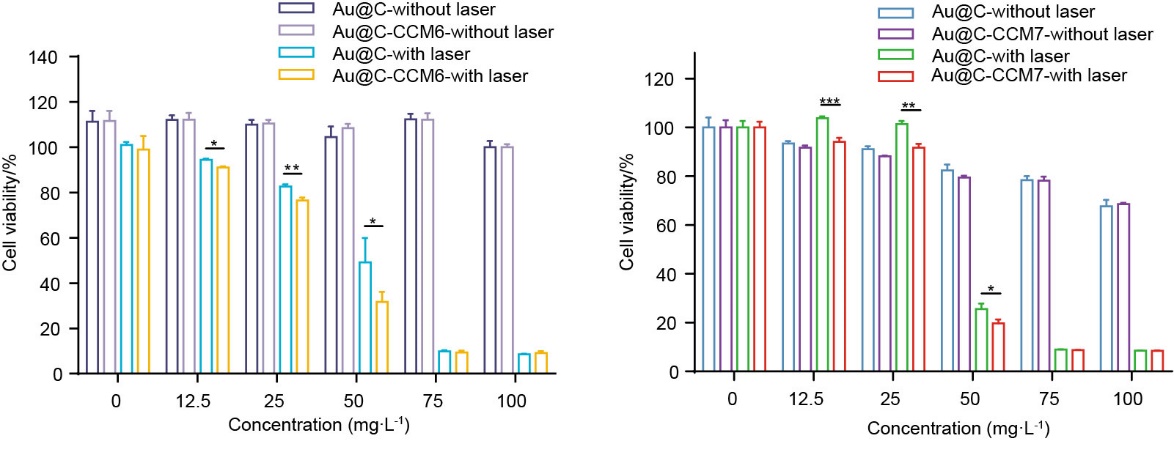
**

**Fig. S9** Viability of HN6 and SCC7 cells in the presence/absence of laser irradiation after 24 h incubation with Au@C and Au@C-CCM at different concentrations (Statistical significance: **P* < 0.05, ***P* < 0.01, ****P* < 0.001).


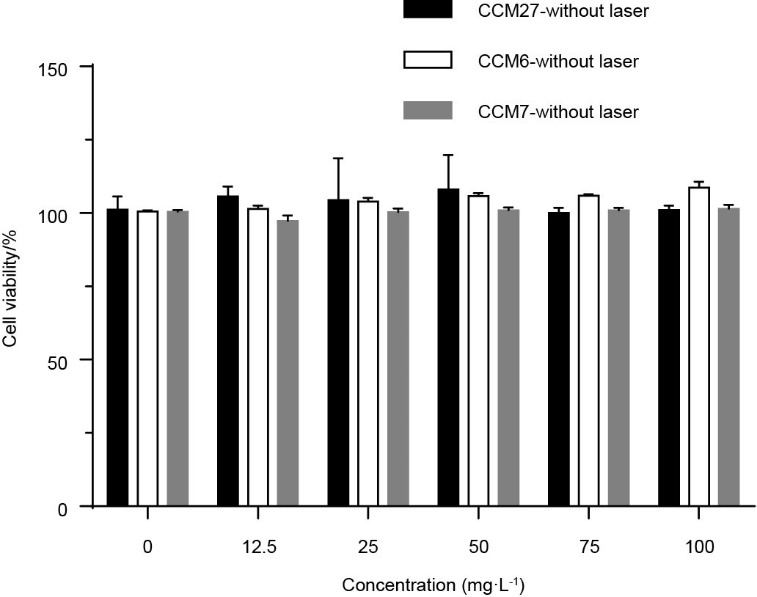


**Fig. S10** Viability of HNSCC cells after 24 h incubation with corresponding pure CCMs at different concentrations.


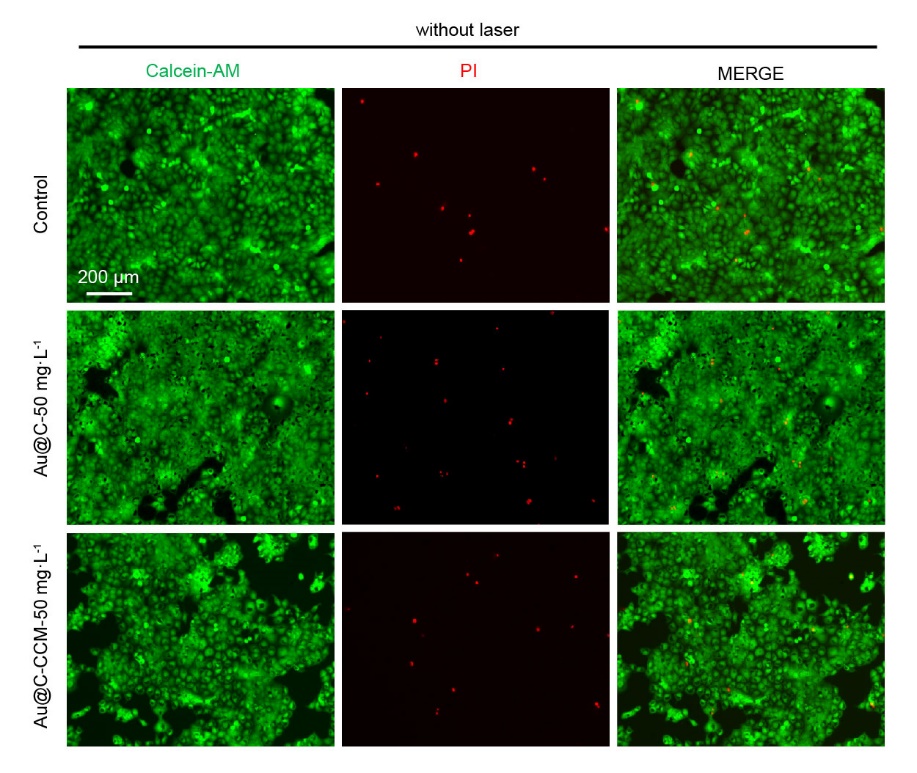


**Fig. S11** Calcein-AM and PI staining of CAL27 cells co-cultured with Au@C or Au@C-CCM without laser irradiation.


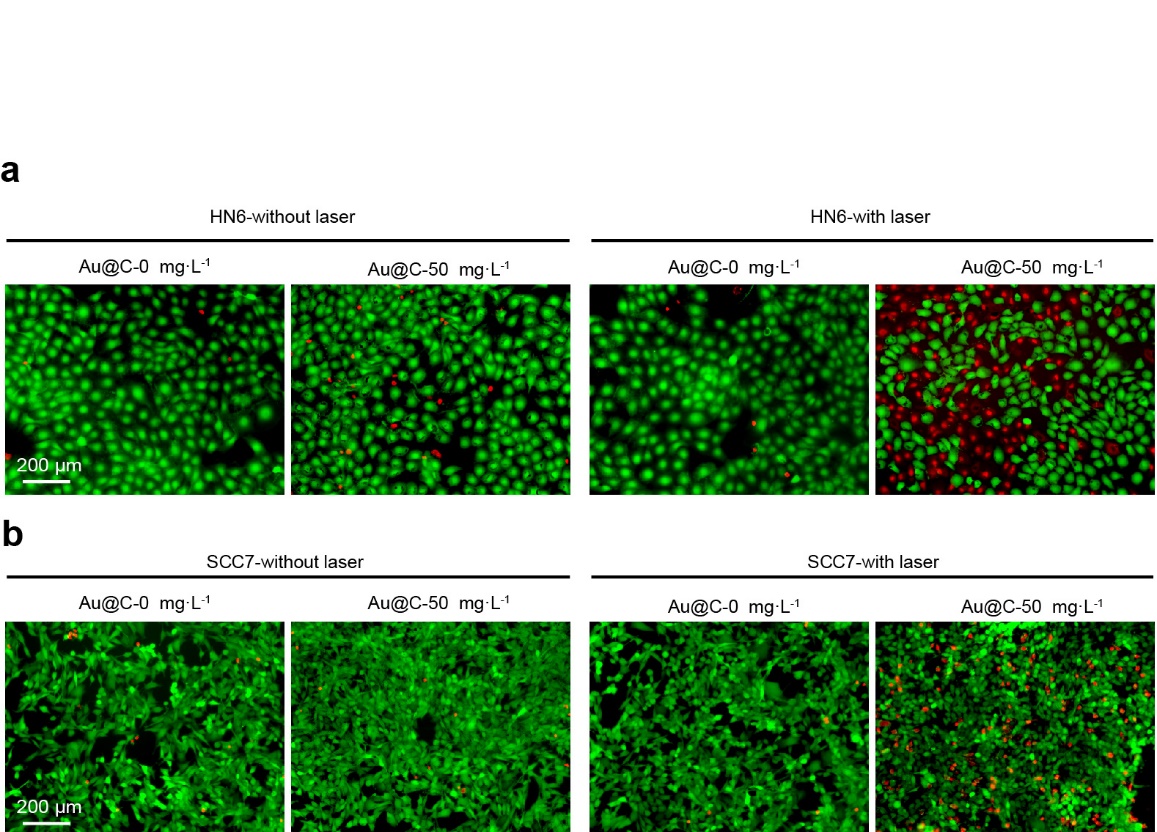


**Fig. S12** Representative Calcein-AM and PI staining images in **a** HN6 and **b** SCC7 cells incubated with Au@C in absence/presence of laser irradiation.


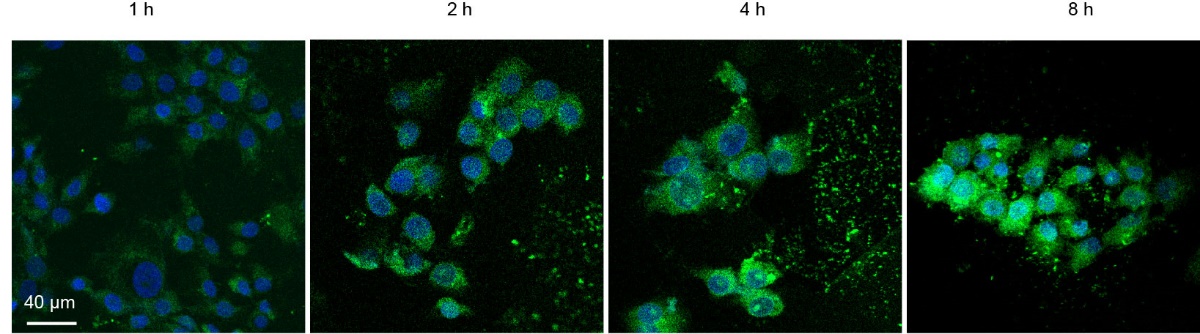


**Fig. S13** Endocytosis experiments of Au@C-CCM27 in CAL27 cells.


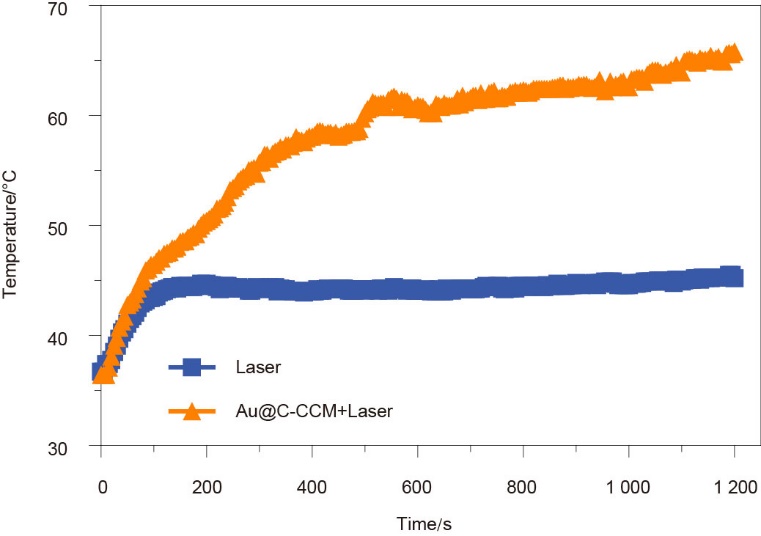


**Fig. S14** Temperature plots of tumor in groups treated with/without Au@C-CCM under 808 nm NIR laser irradiation at various time intervals.


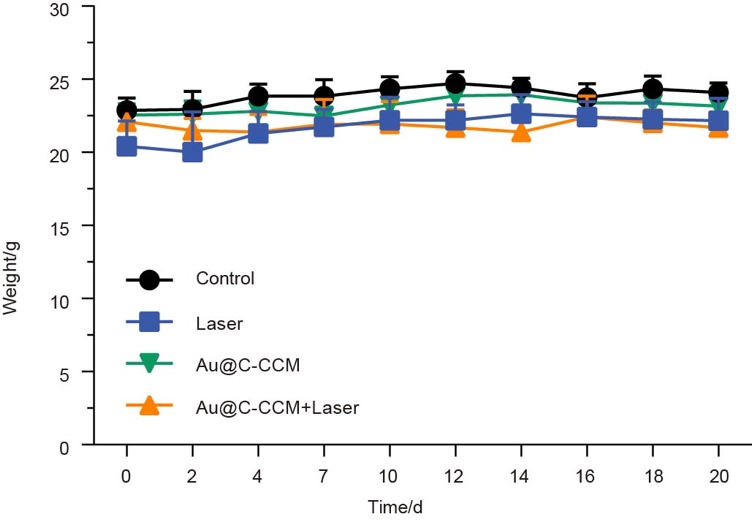


**Fig. S15** Mice body weight change curves following varied treatments (*n*=3).


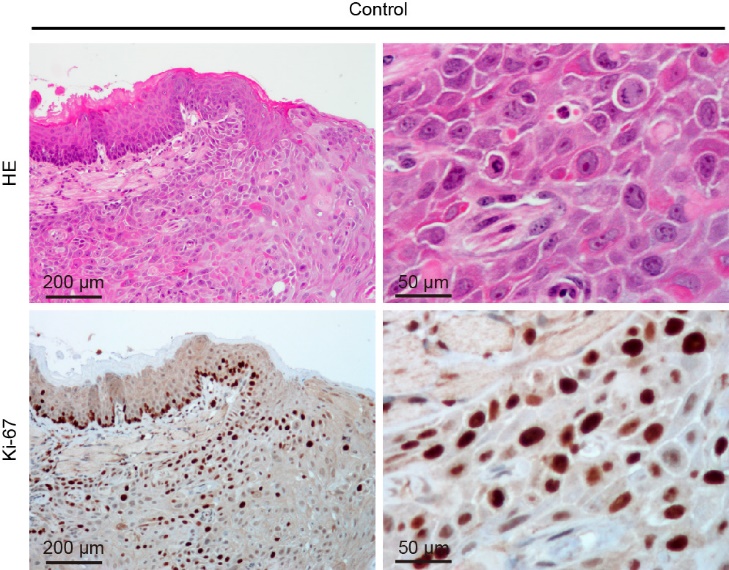


**Fig.** **S16** H&E and Ki-67 staining images of tongue tissue sections from orthotopic tumor model mice without any treatment.


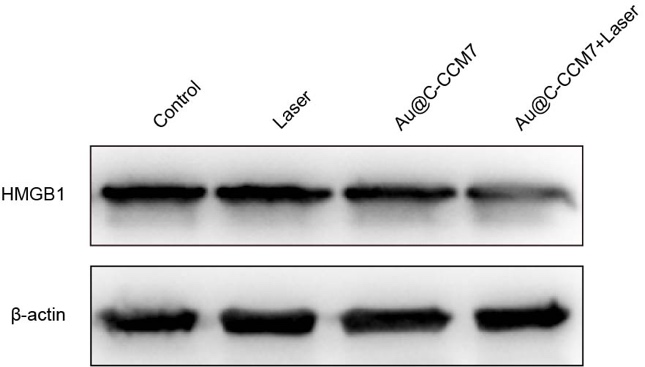


**Fig.** **S17** HMGB1 expression in SCC7 cells after PTT measured by western blot.


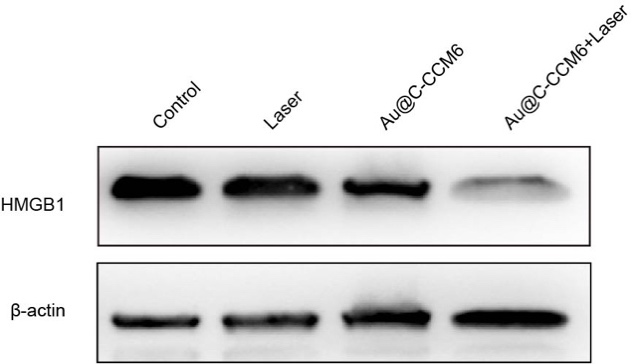


**Fig.** **S18** HMGB1 expression in HN6 cells after PTT measured by western blot.


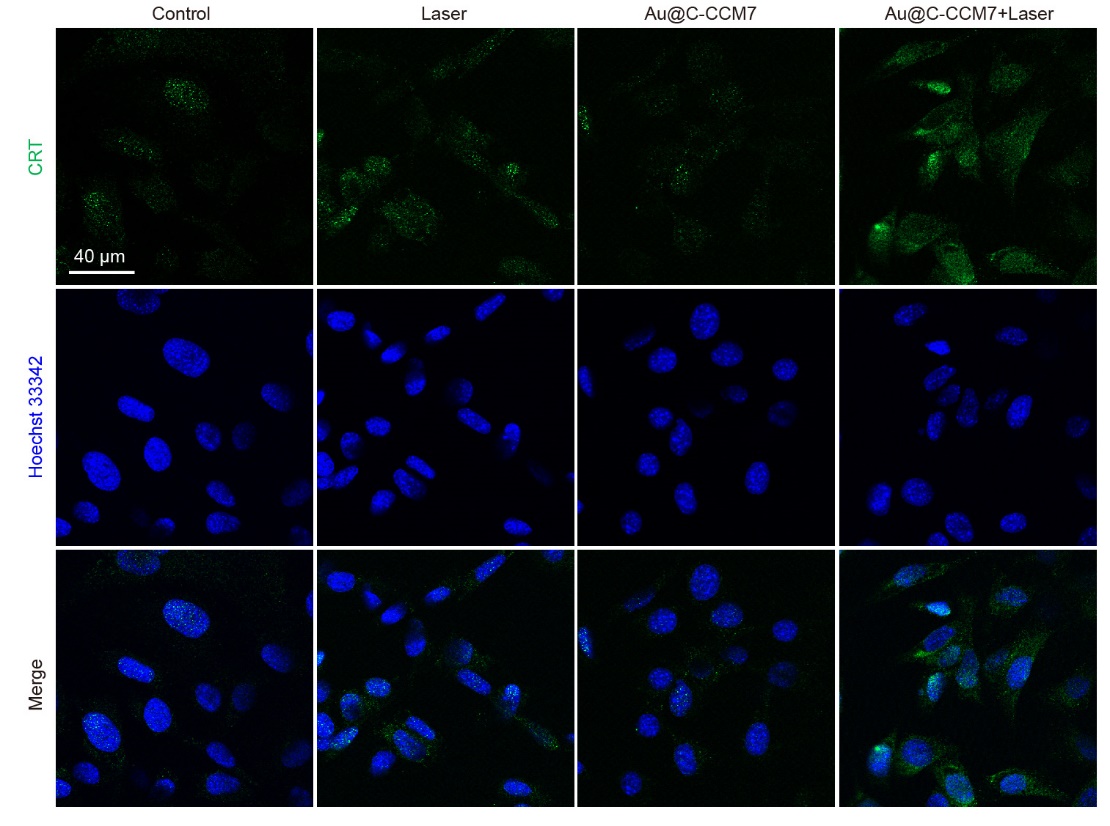


**Fig. S19** Fluorescence images of CRT exposure of SCC7 cells.


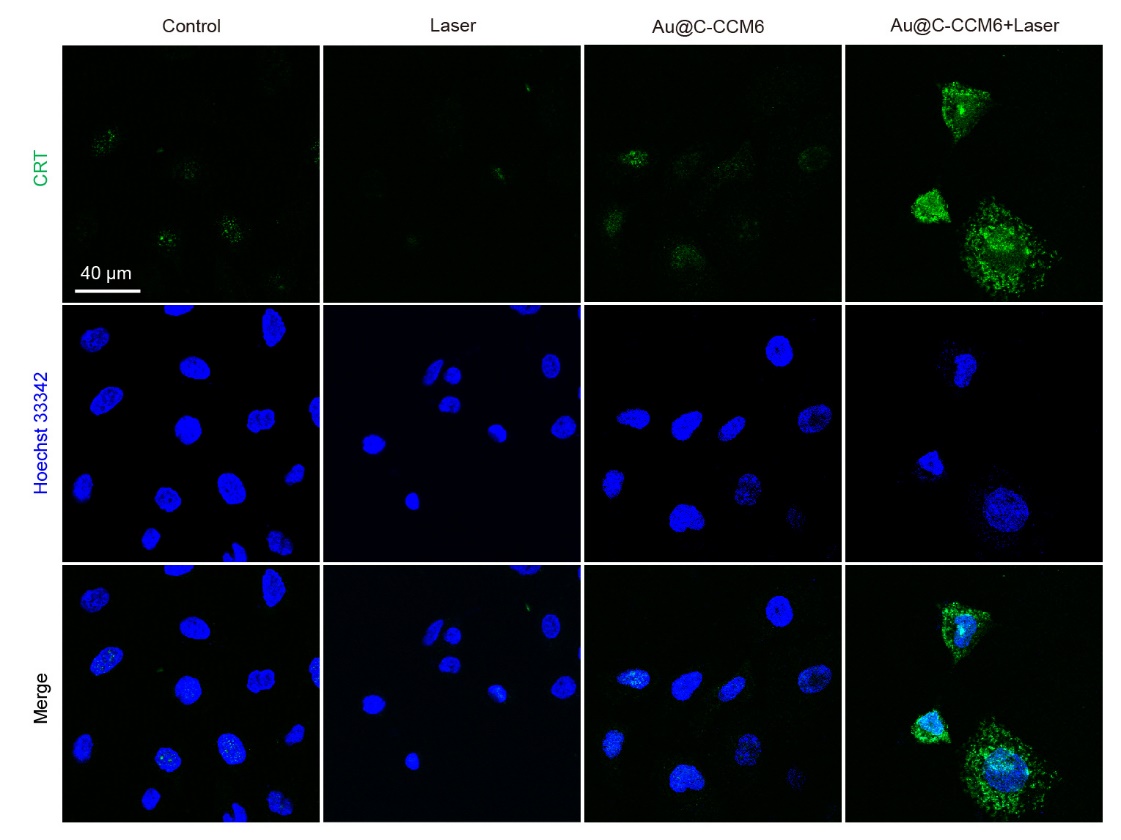


**Fig.** **S20** Fluorescence images of CRT exposure of HN6 cells.

**
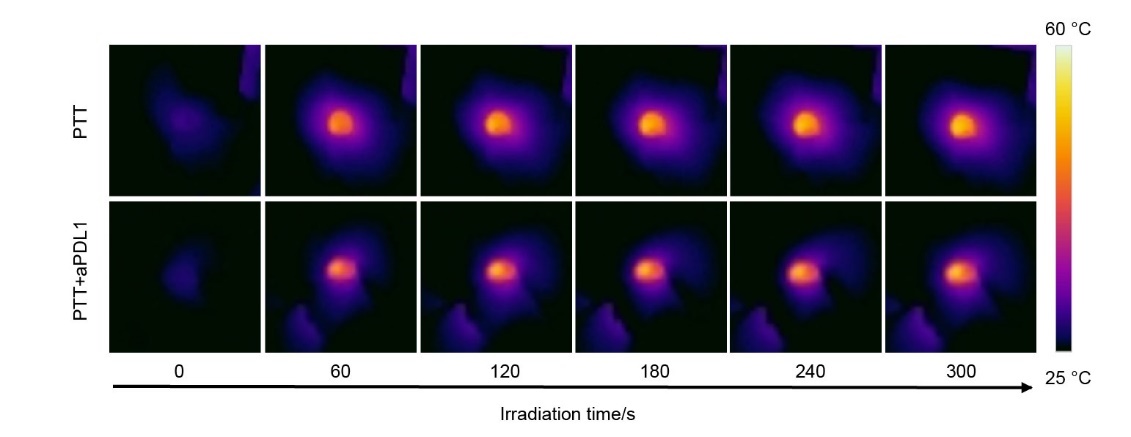
**

**Fig.** **S21** Thermal images of mice in the PTT group and the PTT combined aPDL1 group.

**
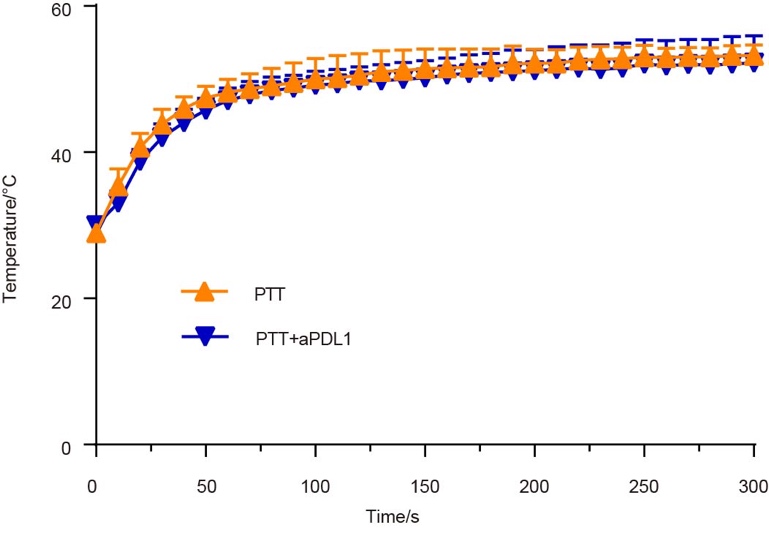
**

**Fig.** **S22** Photothermal heating curves of mice in the PTT group and the PTT combined aPDL1 group.


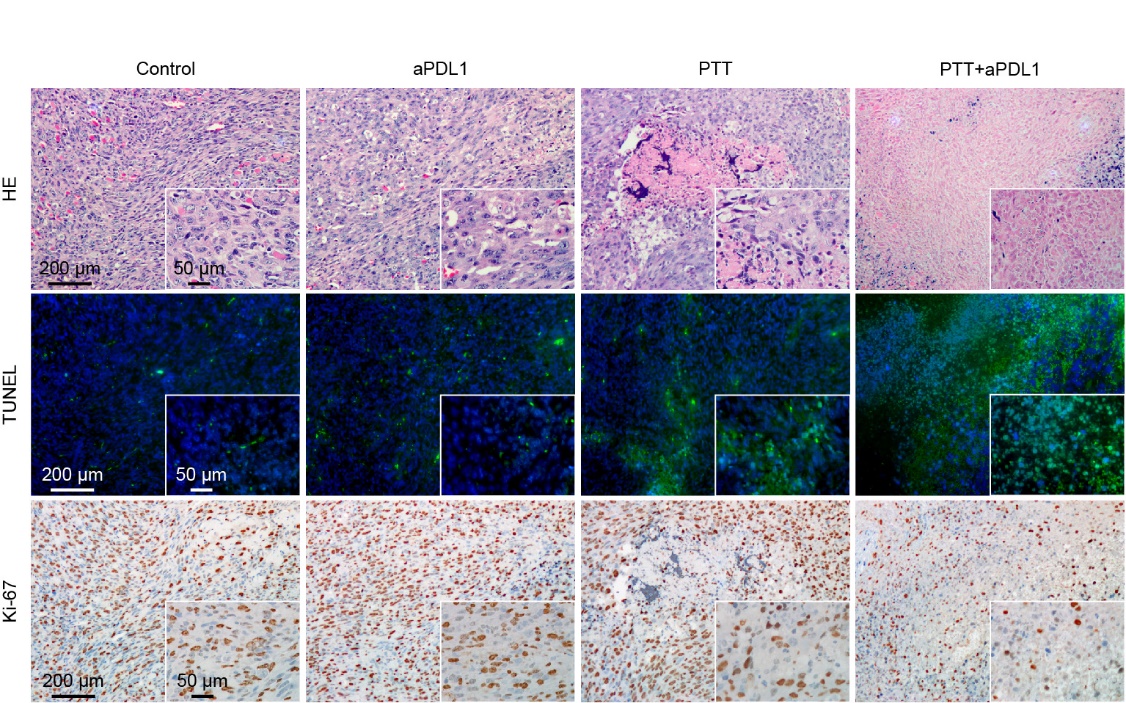


**Fig. S23** Histology slices of H&E, TUNEL, and Ki-67 staining of different groups; inserted in the lower right corner are the magnification images.


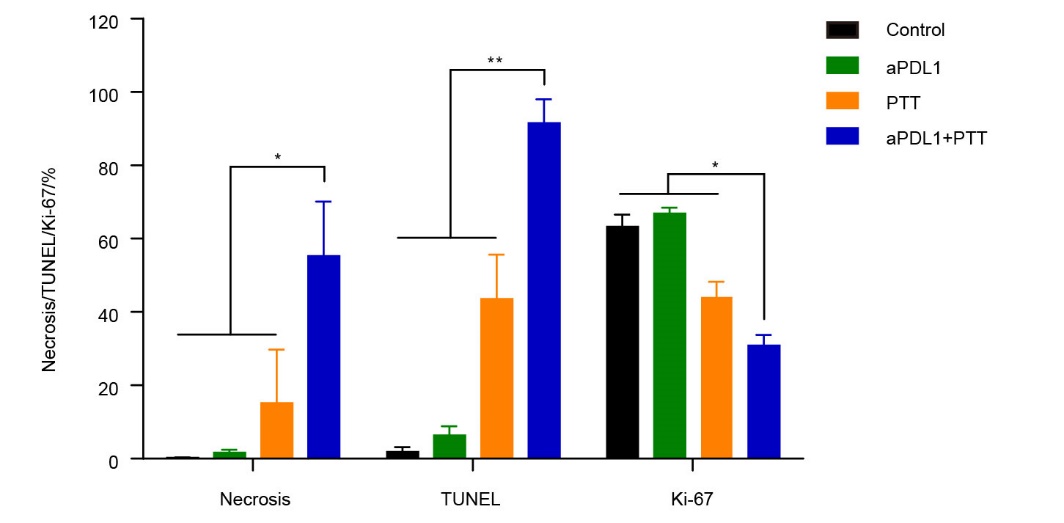


**Fig. S24** Several randomly selected fields of view under 40× microscope were selected for quantitative analysis in each group (Statistical significance: **P* < 0.05, ***P* < 0.01).


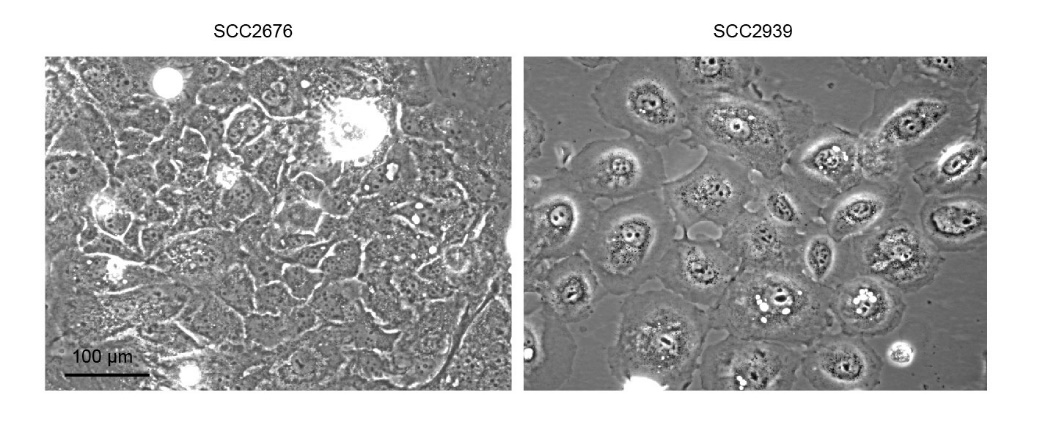


**Fig. S25** PDC morphology with an optical microscope from two HNSCC patients.


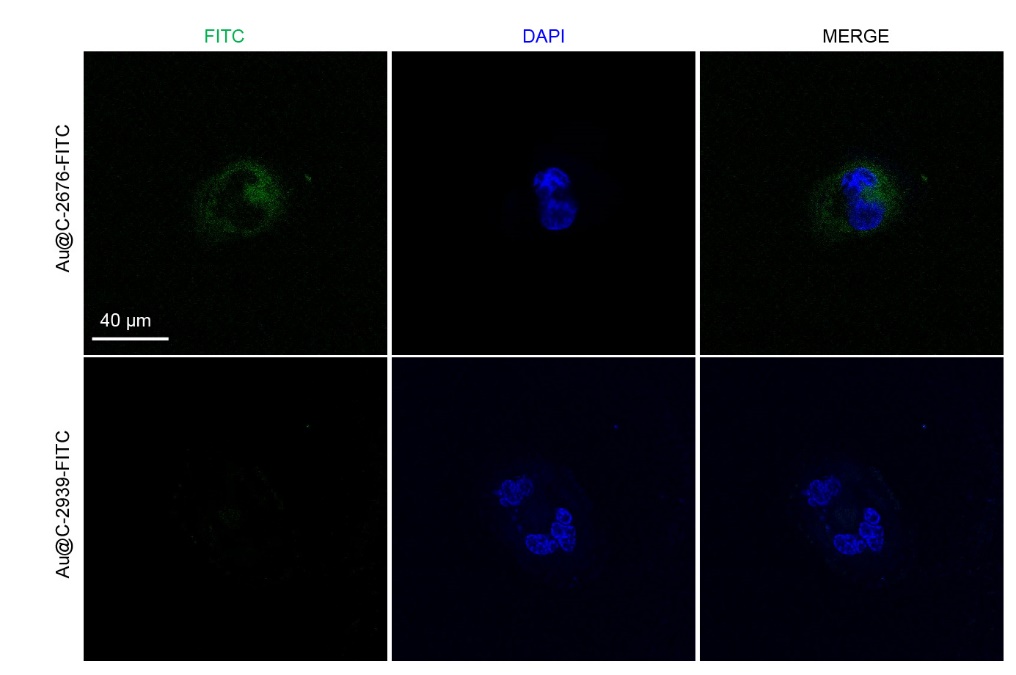


**Fig.** **S26** Cell selectivity of Au@C-2676-FITC compared to Au@C-2939-FITC when cocultured with SCC2676.


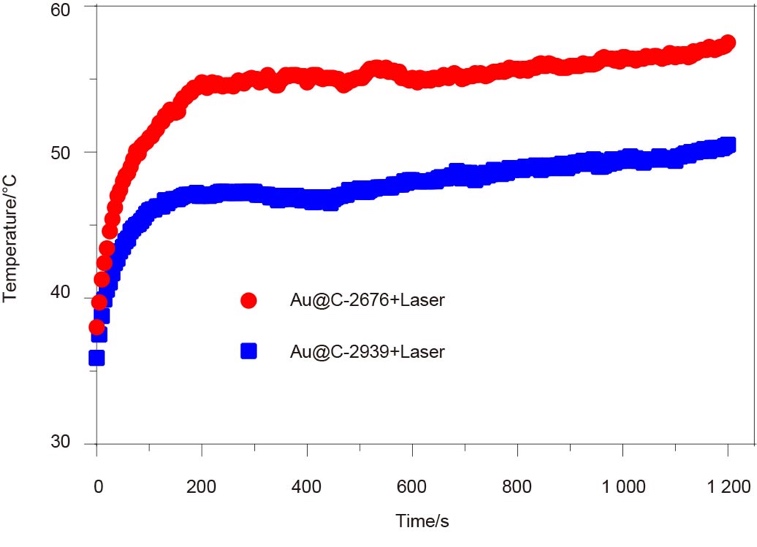


**Fig.** **S27** Temperature plots of the tumor regions in two typical mice from the two groups (Au@C-2676+Laser and Au@C-2939+Laser) during the PT process, respectively.


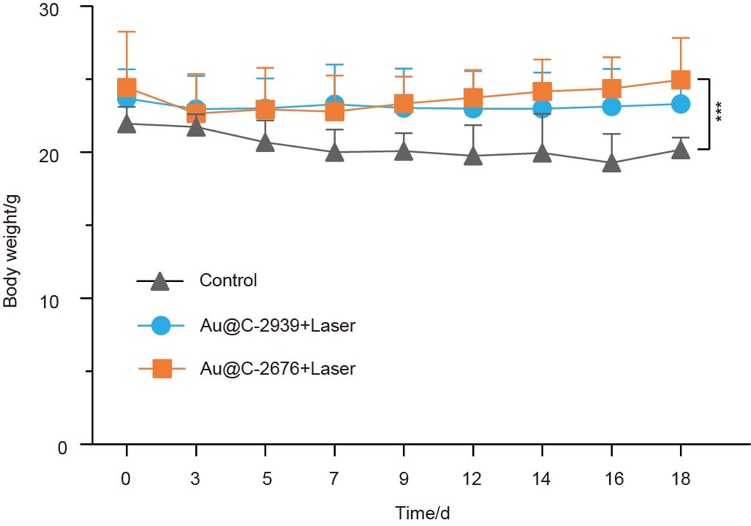


**Fig. S28** Body weight curves after PTT between different groups during the same observation time intervals (*n*=3) (Statistical significance: ****P* < 0.001).


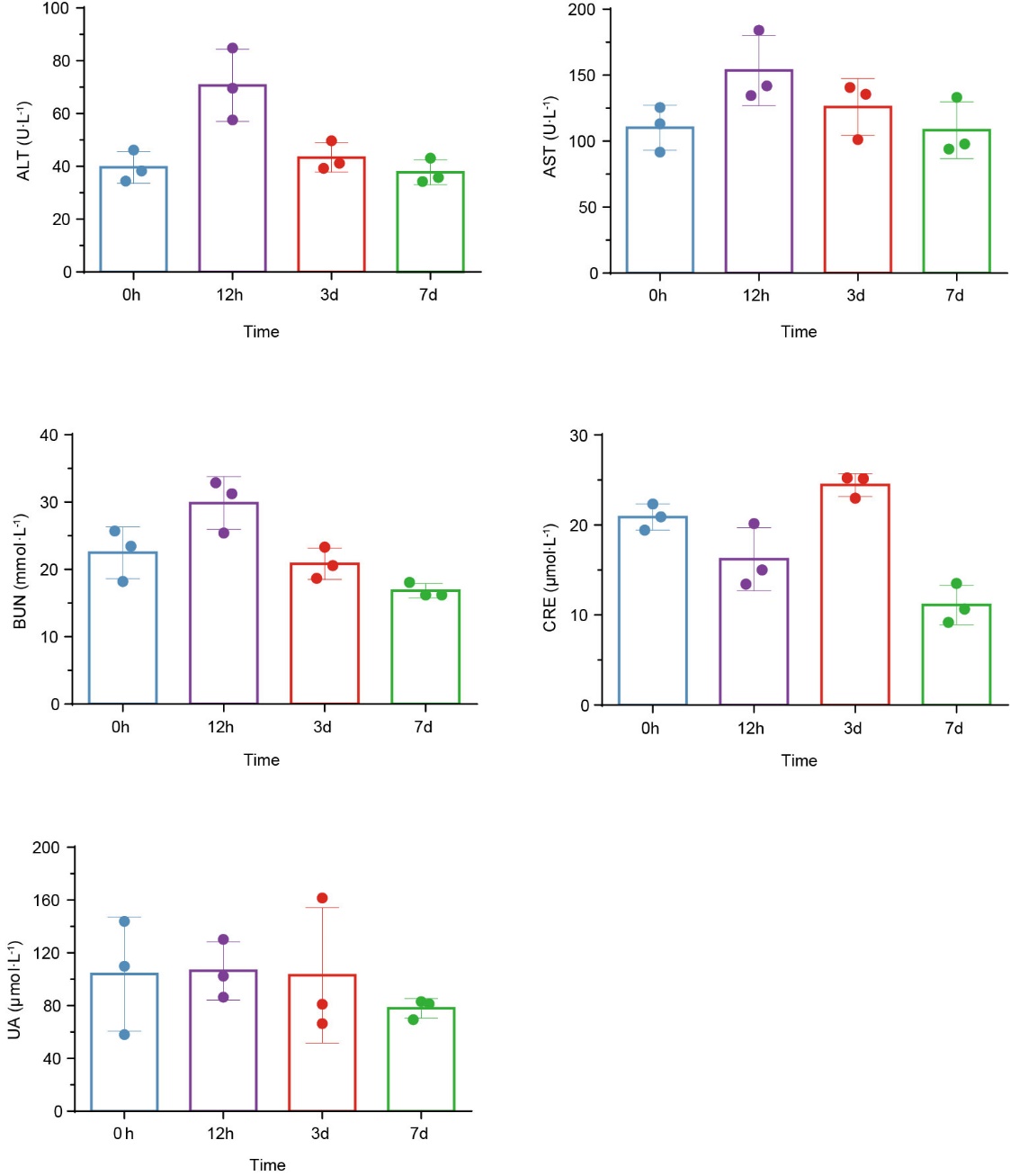


**Fig.** **S29** Blood biochemistry of healthy mice after intravenously injection of Au@C-CCM.


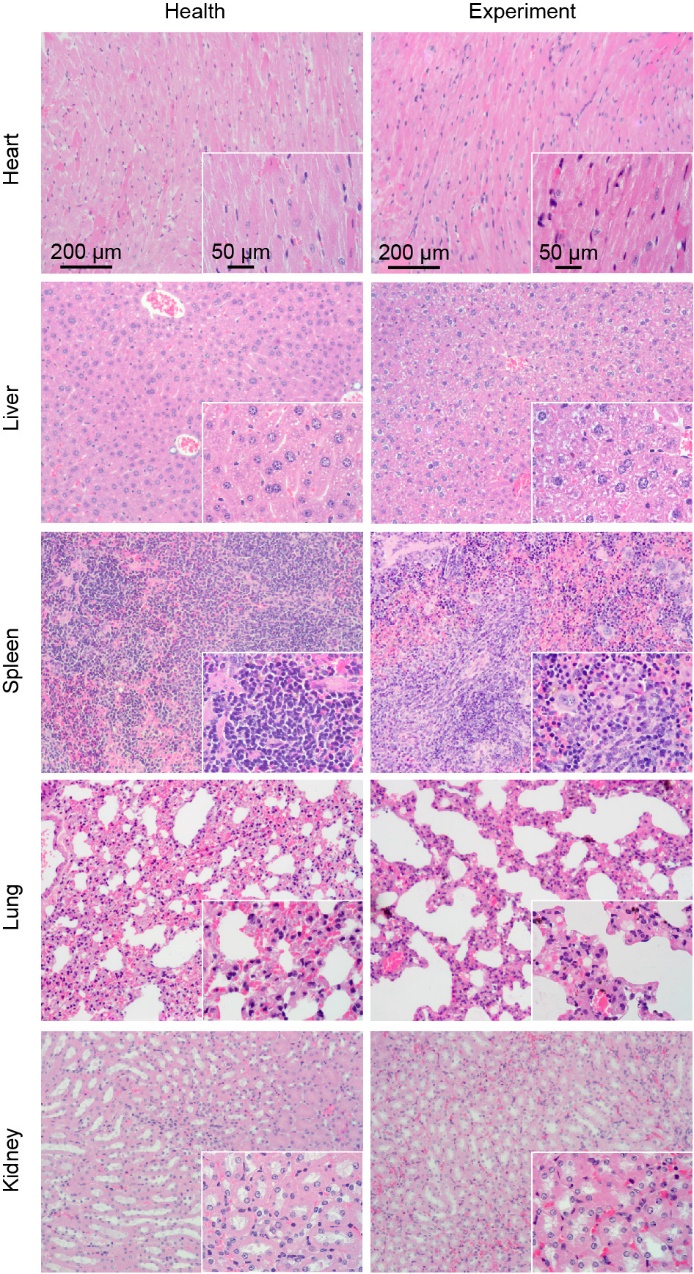


**Fig. S30** H&E staining images of tissue sections from major organs, the corresponding magnification images are inserted in the lower right corner.


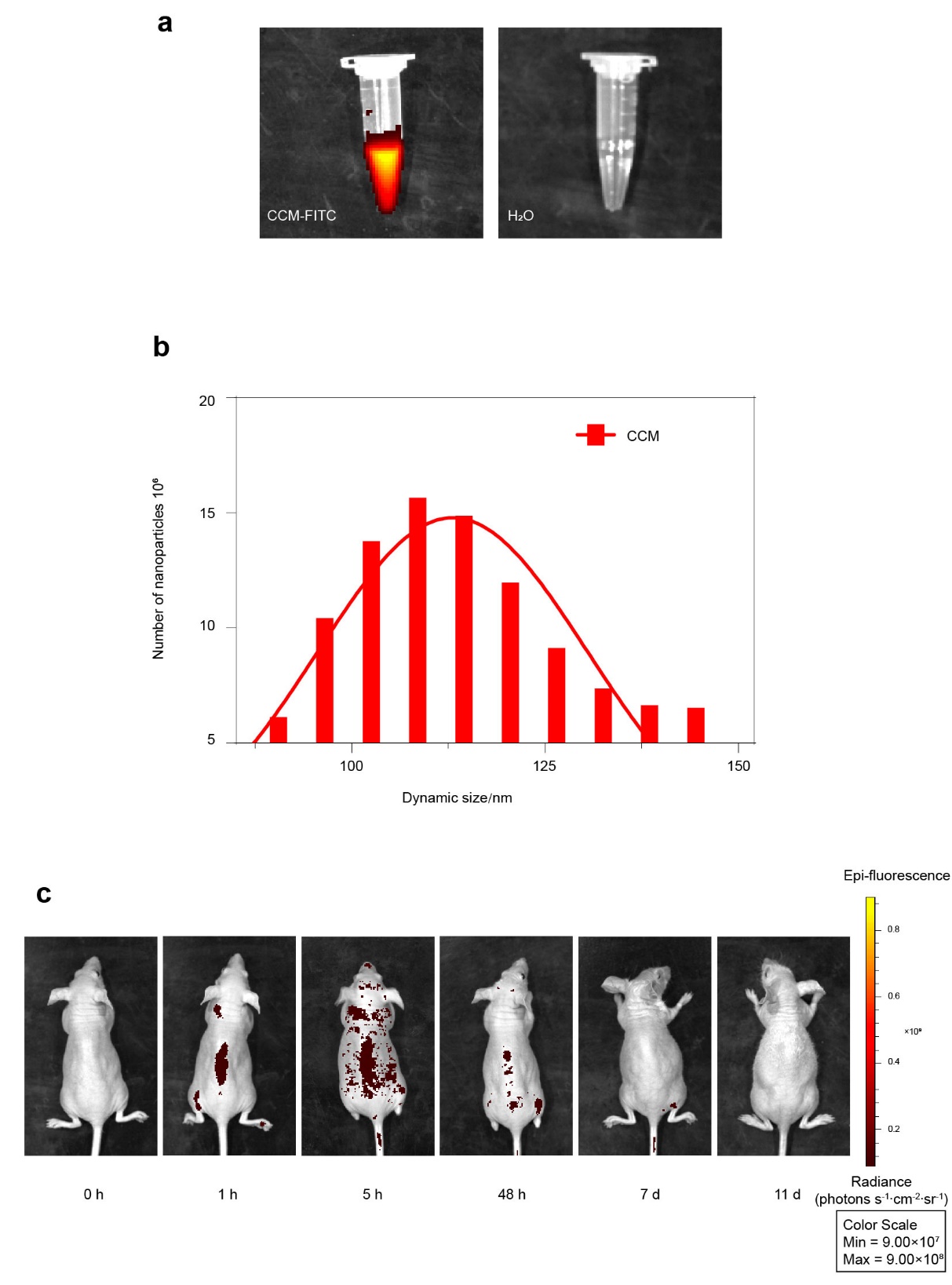


**Fig.** **S31** Biosafety evaluation of CCM in vivo. **a** Fluorescence images of CCM-FITC and water. **b** Dynamic diameter of CCM vesicles. **c** The metabolism of CCM vesicles after intravenously injected within 11 days.

**
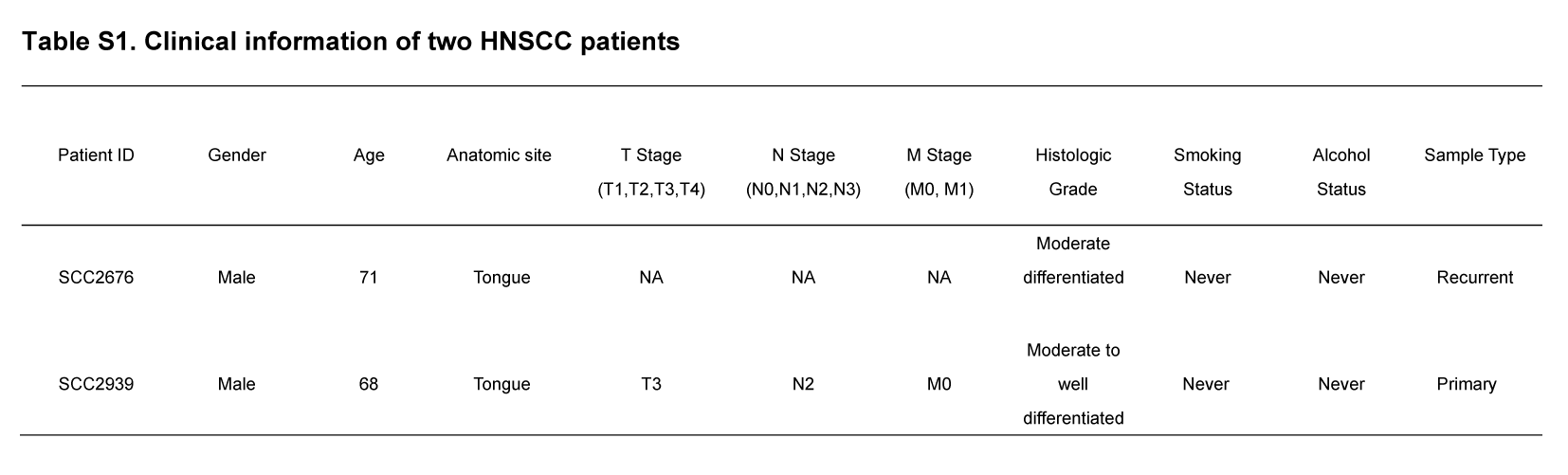
**

**REFERENCES**

1. Roper, D.K., Ahn, W. & Hoepfner, M. Microscale Heat Transfer Transduced by Surface Plasmon Resonant Gold Nanoparticles. *J. Phys. Chem. C. Nanomater. Interfaces* **111**, 3636-3641 (2007).

2. Tian, Q. *et al.* Hydrophilic Cu_9_S_5_ nanocrystals: a photothermal agent with a 25.7% heat conversion efficiency for photothermal ablation of cancer cells in vivo. *ACS Nano* **5**, 9761-9771 (2011).
