# Supplementary material for: A biomimetic nanoplatform for customized photothermal therapy of HNSCC evaluated on patient-derived xenograft models
Source: Int J Oral Sci. 2023 Feb 10;15:9. doi: 10.1038/s41368-022-00211-2 (PMC9918549; doi:10.1038/s41368-022-00211-2)
Supplement: Supplementary file 2 — Supplementary Scheme [file 41368_2022_211_MOESM2_ESM.pdf]

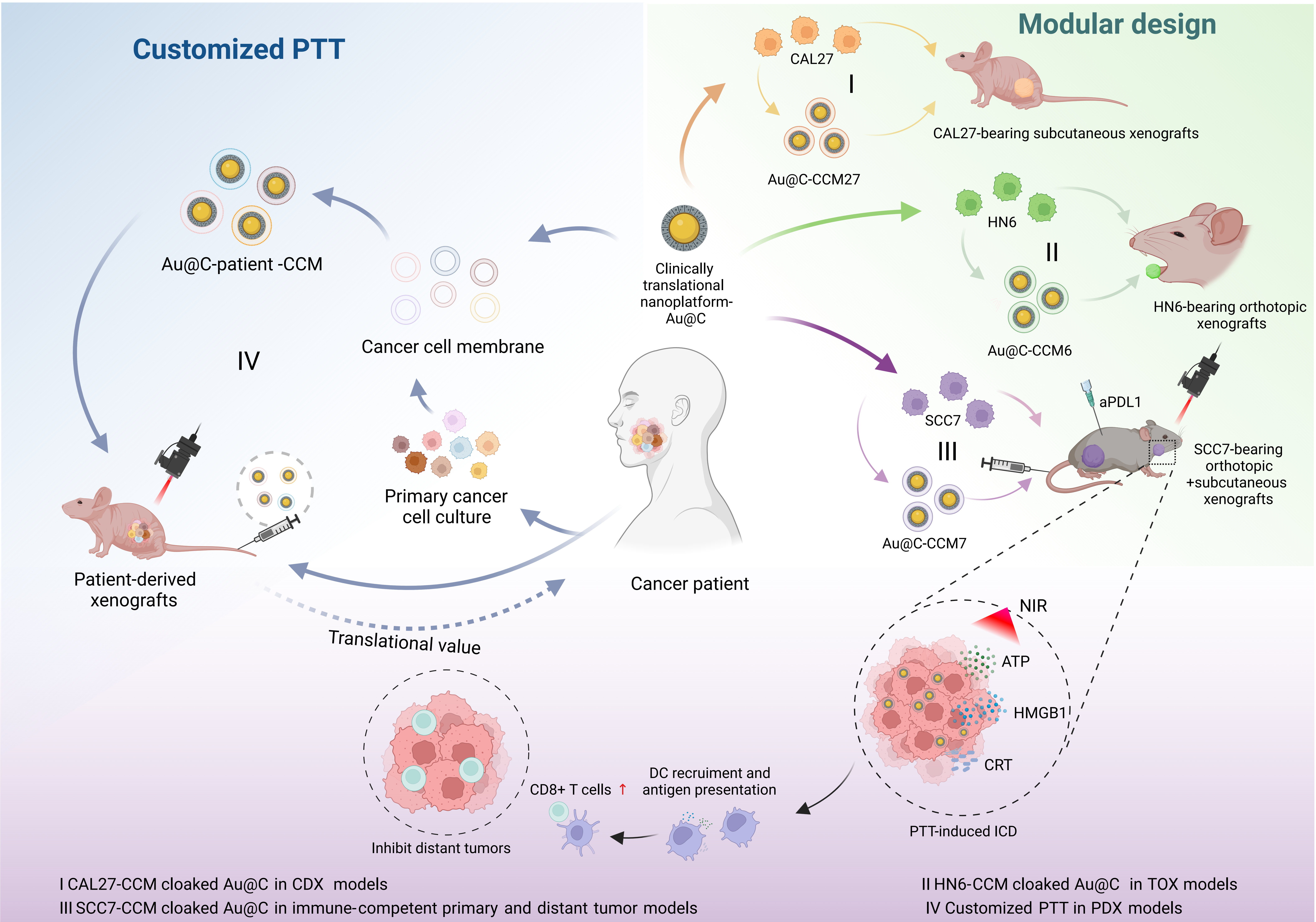
Customized PTT
Modular design
CAL27
I
CAL27-bearing subcutaneous xenografts
Au@C-CCM27
Au@C-patient -CCM
HN6
II
HN6-bearing orthotopic xenografts
Au@C-CCM6
Clinically translational nanoplatform-Au@C
Cancer cell membrane
IV
aPDL1
SCC7
III
SCC7-bearing orthotopic +subcutaneous xenografts
Au@C-CCM7
Primary cancer cell culture
Patient-derived xenografts
Cancer patient
Translational value
NIR
ATP
HMGB1
CRT
PTT-induced ICD
DC recruiment and antigen presentation
CD8+ T cells ↑
Inhibit distant tumors
I CAL27-CCM cloaked Au@C in CDX models
III SCC7-CCM cloaked Au@C in immune-competent primary and distant tumor models
II HN6-CCM cloaked Au@C in TOX models
IV Customized PTT in PDX models

Scheme 1 Schematic illustration of the modular design of the therapeutic processes for customized PTT based on tailored Au@C-CCM NPs.

Schematics were created with BioRender.com
